# SARS-CoV-2 infection of human pluripotent stem cell-derived liver organoids reveals potential mechanisms of liver pathology

**DOI:** 10.1016/j.isci.2022.105146

**Published:** 2022-09-16

**Authors:** Alexsia Richards, Max Friesen, Andrew Khalil, M. Inmaculada Barrasa, Lee Gehrke, Rudolf Jaenisch

**Affiliations:** 1 Whitehead Institute for Biomedical Research, Cambridge, MA 02127, USA; 2 Department of Microbiology, Harvard Medical School, Boston, MA 02115, USA; 3 Wyss Institute for Biologically Inspired Engineering, Harvard University, Boston, MA 02115, USA; 4 John A. Paulson School of Engineering and Applied Sciences, Harvard University, Cambridge, MA 02138, USA; 5 Institute for Medical Engineering and Science, Massachusetts Institute of Technology, Cambridge, MA 02127, USA; 6 Department of Biology, Massachusetts Institute of Technology, Cambridge, MA 02127, USA

**Keywords:** Health sciences, immunology, virology, stem cells research, transcriptomics

## Abstract

Although respiratory symptoms are the most prevalent disease manifestation of infection by Severe Acute Respiratory Syndrome Coronavirus 2 (SARS-CoV-2), infection can also damage other organs, including the brain, gut, and liver. Symptoms of liver damage are observed in nearly half of patients that succumb to severe SARS-CoV-2 infection. Here we use human-induced pluripotent stem cell-derived liver organoids (HLOs) to recapitulate and characterize liver pathology following virus exposure. Utilizing single-cell sequencing technology, we identified robust transcriptomic changes that occur in SARS-CoV-2 infected liver cells as well as uninfected bystander cells. Our results show a significant induction of many inflammatory pathways, including IFN-α, INF-γ, and IL-6 signaling. Our results further identify IL-6 signaling as a potential mechanism for liver-mediated activation of circulating macrophages.

## Highlights

• Stem cell-derived liver organoids (HLOs) are susceptible to SARS-CoV-2 infection
• Within HLOs, hepatocyte-like cells mount the strongest inflammatory response
• Co-culture of HLOs with macrophages increases the inflammatory response to infection
• Infected HLOs secrete IL-6 which promotes macrophage expression of MCP-1

## Graphical abstract

**Figure:**
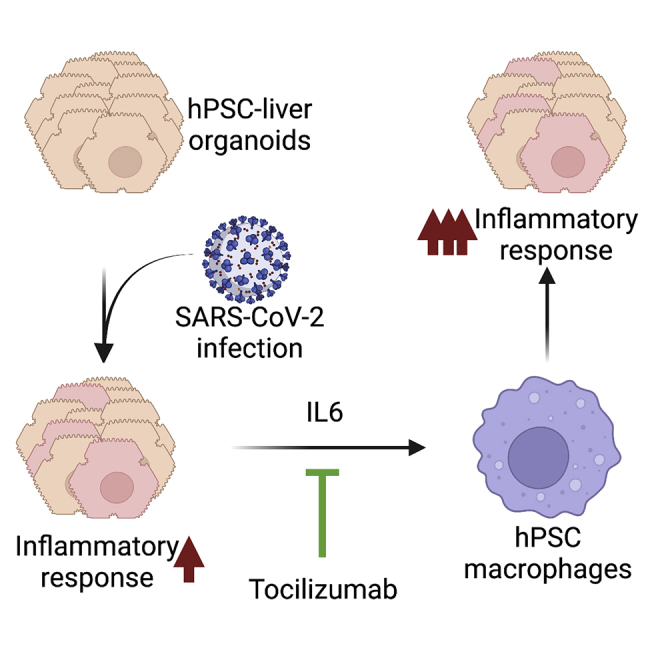

## Introduction

Human coronaviruses have been responsible for severe outbreaks worldwide over the past 20 years, including the Severe Acute Respiratory Syndrome (SARS) outbreak of 2003 and the Middle East Respiratory Syndrome (MERS) outbreak of 2012 (Arora et al., 2020). Most recently, the Severe Acute Respiratory Syndrome Coronavirus 2 (SARS-CoV-2) virus is responsible for a global pandemic leading to over 550 million documented cases and over 6 million deaths as of July 2022 (WHO). Although the primary symptoms of SARS-CoV-2 infection are associated with the respiratory system, there is increasing evidence that severe infection is also associated with liver dysfunction (Lagana et al., 2020; Sonzogni et al., 2020). In one study, approximately 40% of patients with severe SARS-CoV-2 were reported to have liver enzyme abnormalities (Wang et al., 2020). In addition, histopathological examination of liver samples from patients who succumbed to the virus showed evidence of hepatitis and hepatic apoptosis (Lagana et al., 2020; Wang et al., 2020).

An important question is whether the liver damage observed following SARS-CoV-2 infection is the result of the virus infecting liver tissue or if it is the result of the large systemic inflammatory response that often accompanies infection. The primary evidence in support of infection of the liver by SARS-CoV-2 has come from liver autopsy samples from patients who succumbed to SARS-CoV-2 (Lagana et al., 2020; Sonzogni et al., 2020; Wang et al., 2020). Over half of the samples tested were PCR positive for the virus, and imaging of a subset of the samples revealed coronavirus particles in the cytoplasm of hepatocytes (Lagana et al., 2020; Sonzogni et al., 2020; Wang et al., 2020).

Several studies have used single-cell sequencing to identify the specific cell types within the body that express the factors necessary to initiate SARS-CoV-2 infection, specifically ACE2 and TMPRESS2 (Sungnak et al., 2020; Ziegler et al., 2020; Zou et al., 2020). Early reports showed that within the liver, only cholangiocytes, which are the epithelial cells lining the bile ducts within the liver, express significant levels of ACE2 (De Smet et al., 2020; Seow et al., 2021; Sungnak et al., 2020). More recently, a meta-analysis on 31 single-cell sequencing studies also identified epithelial cells within the liver as having high levels of ACE2 and TMPRESS2. However, it was not specified if these were only cholangiocytes (Muus et al., 2021). Although expression of SARS-CoV-2 entry factors may be low in hepatocytes, the liver contains millions of hepatocytes. Therefore, even low expression levels may be sufficient to initiate a substantial infection.

Human pluripotent stem cells (hPSCs) can be differentiated into functional human cells and assembled into organoids which can be used for modeling human disease. Previous studies using hPSC-derived liver organoids showed that ACE2 was expressed in the majority of hepatocytes (Yang et al., 2020). These studies further showed that hPSC-derived liver organoids were susceptible to SARS-CoV-2 infection and showed strong induction of inflammatory cytokines and chemokines (Yang et al., 2020). Importantly, the analysis performed in this report relied on bulk RNA sequencing of the entire organoid. Therefore, the identification of the specific transcriptional signatures associated with infected cells within the organoid was not possible.

In this report, we use hPSC technology to generate human liver organoids. Using these organoids as a model system for SARS-CoV-2 liver infection, we employed single-cell sequencing to identify the cell-intrinsic responses of liver cells to SARS-CoV-2 infection. Our results identify inflammatory pathways activated in the liver cells that may be responsible for hepatitis associated with severe SARS-CoV-2 infection. This system provides a physiologically relevant platform for further studies on drug development and modeling viral pathogenesis.

## Results

### Human pluripotent stem cell-derived liver organoids are susceptible to SARS-CoV-2 infection

**Figure 1:**
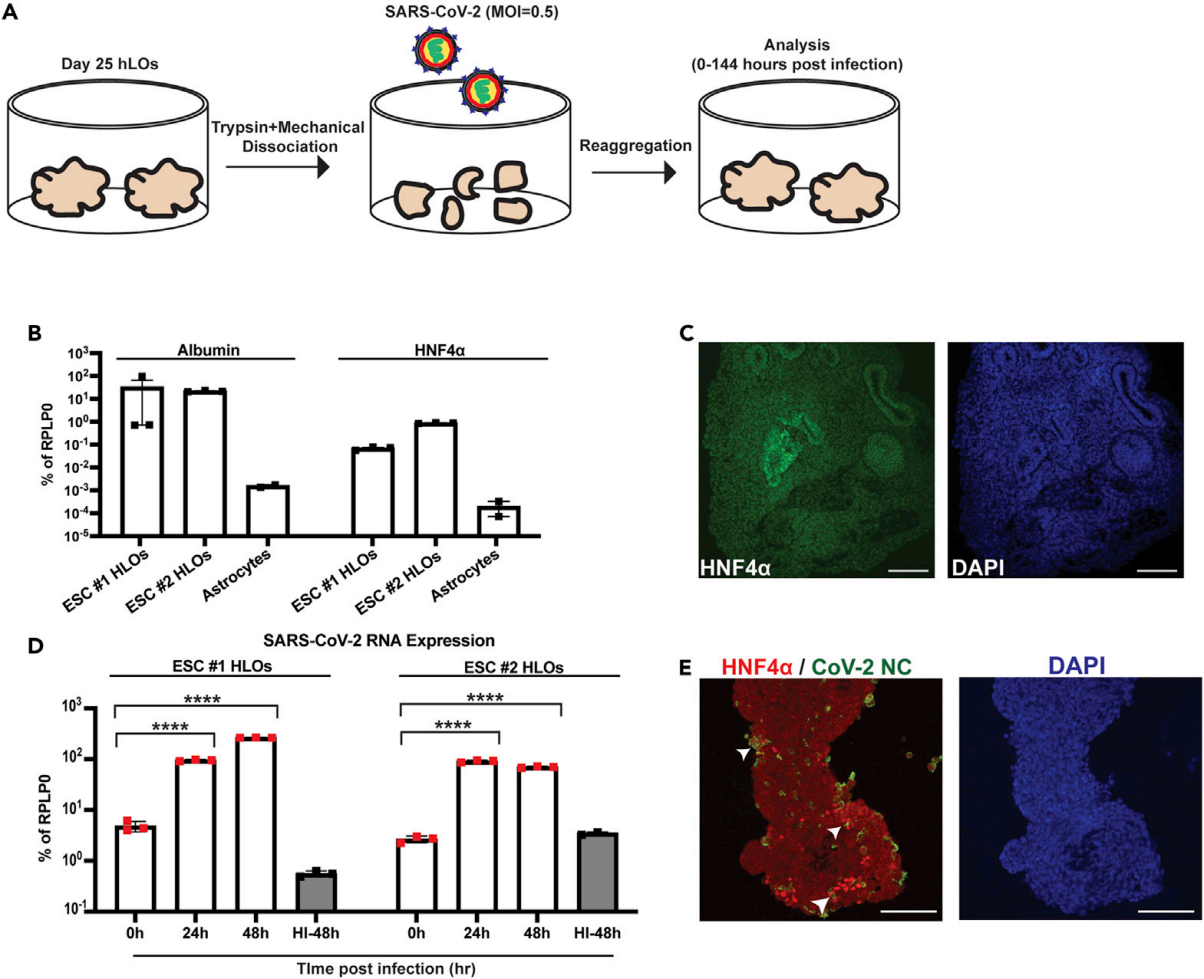
hPSC-derived liver organoids are susceptible to SARS-CoV-2 infection (A) Experimental schematic of infection of hPSC-derived liver organoids (HLOs) with SARS-CoV-2. (B) RT-qPCR of liver-specific genes in HLOs derived from either H1 ESCs or Allen ESCs. Astrocytes derived from H1 ESCs were included as a negative control. All samples were plated in triplicate for RT-qPCR. (C) Representative confocal image of HLO stained for HNF4α; scale bars, 100 μm. (D) RT-qPCR of SARS-CoV-2 nucleocapsid (NC) expression in infected HLOs at 0, 24, and 48 h post-infection (hpi). HI-48 = HLOs infected with heat-inactivated SARS-CoV-2 for 48 h. All samples were plated in triplicate for RT-qPCR. (E) Representative confocal images of HLOs infected with SARS-CoV-2 and stained for SARS-CoV-2 nucleocapsid (NC) and HNF4α; scale bars, 100 μm. For (B) and (D) values are expressed as mean ± SEM (∗∗∗∗ = p < 0.0001 based on two-tailed unpaired *t* test).

To assess the potential of hepatocytes to be infected by SARS-CoV-2, we differentiated hPSCs from multiple donors into liver organoids using a previously published protocol and exposed them to the virus (Figure 1A) (Ouchi et al., 2019). hPSC-derived liver organoids (HLOs) expressed the hepatocyte-specific markers HNF4a and albumin, as well as the epithelial marker cytokeratin 17 (KRT17; Figures 1B, 1C, and S1B). These epithelial cells likely represent a population of cholangiocyte-like cells. Cholangiocytes are epithelial cells that line bile ducts AND have been shown to present in HLOs (Ouchi et al., 2019).

To recapitulate the infection of the cells at the luminal surface of the liver, HLOs were dissociated into small fragments prior to infection with SARS-CoV-2. As a negative control, HLO fragments were incubated with heat-inactivated SARS-CoV-2 at a matched MOI. Heat-inactivation has previously been shown to disrupt the SARS-CoV-2 virion resulting in non-infectious virus (Loveday et al., 2021). Following incubation with SARS-CoV-2, HLO fragments were re-aggregated to generate infected HLOs. SARS-CoV-2 successfully infected HLOs as evidenced by increasing viral RNA load over the course of the infection (Figure 1D). In addition, viral nucleocapsid (NC) staining was detected in HNF4α positive cells at 48 h post-infection (Figure 1E). Viral replication was also observed in cholangiocyte-like cells as indicated by the presence of double-stranded RNA (dsRNA) which is generated as part of SARS-CoV-2 genome replication (Figure S1B). To confirm the production of infectious virus in HLO cells, HLOs were dissociated and plated in 2D. Cells were infected with SARS-CoV-2 and media was collected over the course of infection. A significant release of infectious SARS-CoV-2 was observed from 0 to 24 and 24 to 48 h post-infection with a reduction in infectious virus production from 48 to 72 h post-infection (Figure S1A).

### Characterization of the transcriptomic response of human-induced pluripotent stem cell-derived liver organoids to SARS-CoV-2 infection

**Figure 2:**
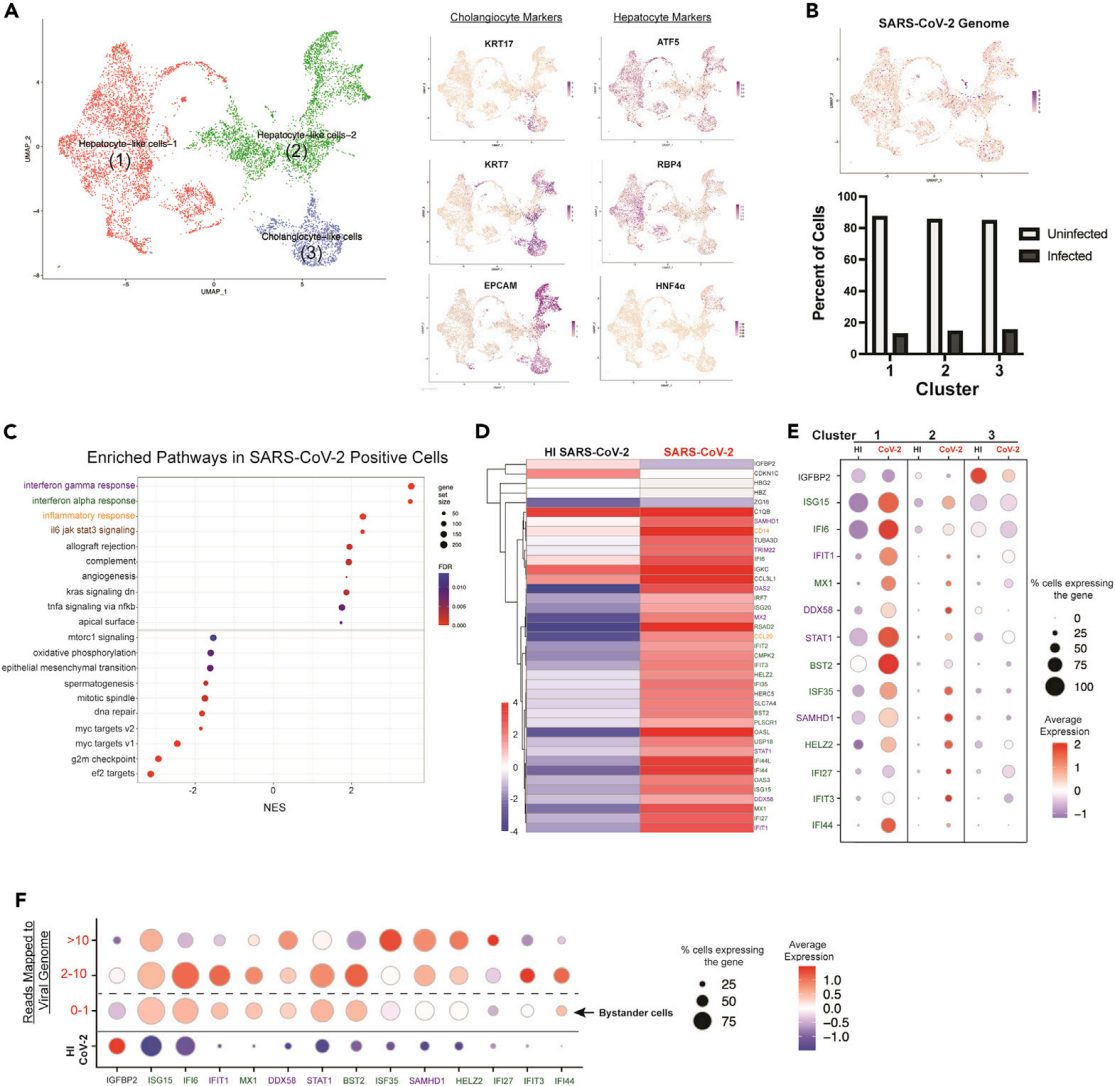
Transcriptomic response of HLOs to SARS-CoV-2 infection. HLOs were infected with SARS-CoV-2 and sequenced with 10x single-cell sequencing (A) The three major clusters were identified as (1) Hepatocyte-like cells 1 (ATF5 & RBP4 positive) (2) Hepatocyte-like cells 2 (positive for both hepatocyte and cholangiocyte markers) and (3) Cholangiocyte-like cells (KRT7, KRT17 & EPCAM positive). (B) Projection of cells with SARS-CoV-2 transcripts onto the UMAP. The graph below shows the percentage of cells with at least one read mapping to the SARS-CoV-2 genome (infected) in each cluster. (C) Gene set enrichment analysis (GSEA) showing the top upregulated and downregulated gene sets in infected HLO cells versus cells in HLOs exposed to HI SARS-CoV-2. (D) Heatmap of all significantly differentially expressed genes (Adj. p value < 0.05) in infected HLO cells compared to cells in HLOs exposed to HI SARS-CoV-2. Differential expression was conducted on aggregated counts of all the cells in each condition and included HLOs generated from H1 ESCs and 1016 IPSCs. Color of gene name corresponds to associated gene set (purple = IFNγ, green = IFNα, orange = inflammatory response). (E) Dot-plot showing expression of top differentially expressed genes from D - that are expressed in at least 50% of one of the conditions plotted-in each major cluster in infected HLO cells versus cells in HLOs exposed to HI SARS-CoV-2. (F) Expression pattern of top differentially expressed genes showed in E in relation to the number of SARS-CoV-2 genomes detected in the cell. For panels (A–C), data are from HLOs generated from H1 ESCs and 1016 IPSCs. For panels (D–F), data are from HLOs generated from H1 ESCs.

To identify the transcriptional changes induced by SARS-CoV-2 infection, we performed single-cell RNA sequencing at 48 h post-infection. Three major cell clusters were identified based on the expression of hepatocyte and epithelial cell markers (Figure 2A). The cluster labeled *Cholangiocyte-like cells* expressed high levels of the epithelial markers KRT17, KRT7, and EPCAM. Two clusters of hepatocyte-like cells were identified. *Hepatocyte-like cells 1* expressed the hepatocyte-specific markers HNF4α, ATF5, and RBP4. *Hepatocyte-like cells 2* expressed markers of both hepatocytes and epithelial cells and may represent cells in a state of de-differentiation owing to the cellular stress induced by the re-aggregation procedure (Figures 2A and S2). Integration of our data with single nuclei sequencing from SARS-CoV-2 infected patients showed that the transcriptional profile of cholangiocyte-like cells from HLOs was similar to primary cholangiocytes, whereas hepatocyte-like cells from HLOs were more similar to primary hepatocytes (Figures S3A and S3B).

For our analysis cells were considered infected if they had more than one read mapping to the SARS-CoV-2 genome. This threshold was selected based on the finding that very few cells in HLOs exposed to heat-inactivated SARS-CoV-2 displayed more than one read mapping to the SARS-CoV-2 genome (Table S1). The reads mapping to the SARS-CoV-2 genome on cells in the HI samples could be owing to the input heat-inactivated virus attaching to HLO cells. In HLOs exposed to SARS-CoV-2 infected cells were detected in all three cell types at approximately equal frequencies (Figure 2B and Table S2).

The gene expression patterns of infected cells within HLOs exposed to live SARS-CoV-2 were compared with cells within HLOs exposed to heat-inactivated SARS-CoV-2. Gene Set Enrichment Analysis (GSEA) revealed significant upregulation of inflammatory pathways, including interferon-alpha (IFNα) response, interferon gamma (IFNγ) response, and interleukin-6 (IL-6) signaling in SARS-CoV-2 infected cells (Figure 2C). Genes where the differential expression analysis showed an adjusted p value of <0.05 were considered to be significantly upregulated or downregulated. Further analysis of significantly differentially expressed genes showed increased expression of many IFN-stimulated genes including IFIT1, IFI27, MX1, and OAS3 (Figures 2D and S4A).

To determine the contribution of the different cell types within the HLO to the inflammatory response induced by SARS-CoV-2 infection, the expression of significantly changed genes was separately examined in each cluster. Infection of hepatocyte-like cells resulted in a greater increase in expression of many inflammatory genes as compared to the infection of cholangiocyte-like cells (Figures 2E and S4B). In support of this finding, in SARS-CoV-2-infected patients, expression of the inflammatory genes BST2, IFI6, IFI35, and ISG15, was higher in hepatocytes relative to cholangiocytes (Figure S3C). Through single-cell sequencing, we were able to group infected cells within HLOs based on viral RNA content. Cells within HLOs were divided into three groups based on the number of reads mapping to the SARS-CoV-2 genome and compared to cells within HLOs exposed to heat-inactivated SARS-CoV-2 (Figures 2F, and S4C; Table S1). The expression of the top differentially expressed genes (Figure 2D), revealed the induction of inflammatory signaling in cells that have no detectable viral infection but reside in HLOs that were exposed to live virus (Figures 2F, S4C, and S5). In addition, several key inflammatory markers including (IFI6, IFIT1, and BST2) show reduced expression in cells with greater than 10 reads mapping to the viral genome (Figure 2F). These results suggest that at high levels of infection, the virus may dampen the expression of genes in many inflammatory pathways. Alternatively, cells with high expression of inflammatory genes may more effectively restrict viral infection. To determine if exposure to viral particles is sufficient to induce an inflammatory response, or if viral replication is necessary, HLOs were exposed to either live SARS-CoV-2, heat-inactivated SARS-CoV-2, or mock infected for either 48 or 120 h and total RNA extracted for bulk RNA sequencing (Figure S6A). At 48 h post-infection exposure to live or heat-inactivated SARS-CoV-2 had similar effects on the expression of many of the inflammatory genes we detected in our single-cell sequencing data. However, by 120 h post-infection, live SARS-CoV-2 induced significantly higher expression of the majority of these genes compared to heat-inactivated virus. These data suggest that at 48 h post-infection the majority of the overall inflammatory response may be owing to exposure to the viral antigens rather than viral replication in infected cells. Single-cell sequencing was able to detect the changes in transcription that are unique to infected cells which make up only a fraction of the total cells (Figure 2B). Interestingly, there was very little overlap in the most upregulated genes induced by live SARS-CoV-2 or heat-inactivated SARS-CoV-2 at 120 h post-infection (Figures S6B and S6C).

### SARS-CoV-2 infection of human-induced pluripotent stem cell-derived liver organoids promotes the secretion of IL-6

**Figure 3:**
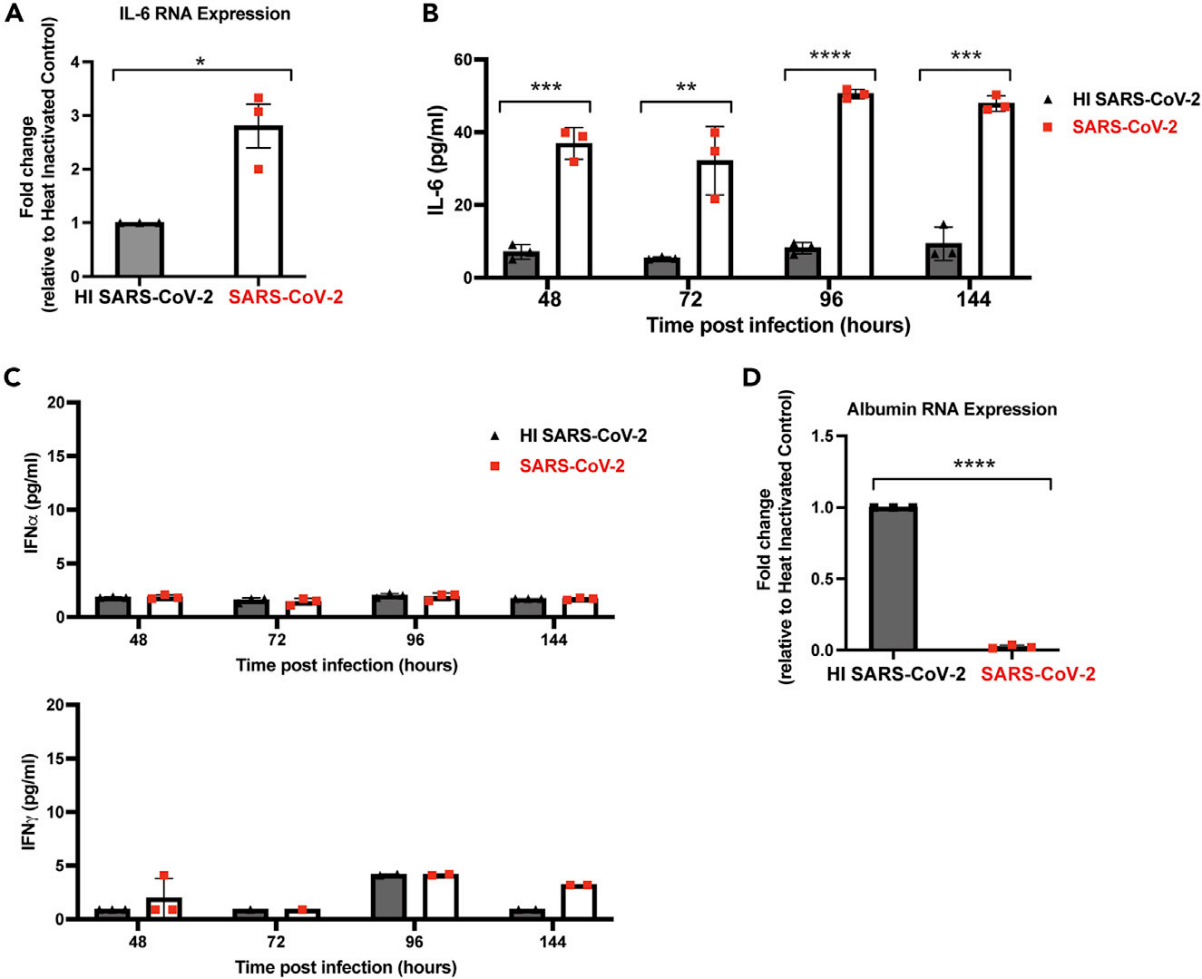
SARS-CoV-2 infection of HLOs promotes the secretion of IL-6 but not IFNα or IFNγ (A) RT-qPCR was performed on HLOs infected with either live SARS-CoV2 or HI SARS-CoV2 at 144 h post-infection to look at IL-6 expression. Samples were plated in triplicate for RT-qPCR. (B and C) Luminex analysis on media from infected cultures was performed at the indicated time post-infection (n = 3). Target is indicated on the y axis. Values are expressed as mean ± SD (∗∗ = p < 0.01; ∗∗∗ = p < 0.001; ∗∗∗∗ = p < 0.0001; based on two-tailed unpaired *t* test). (D) RT-qPCR was performed on HLOs infected with either live SARS-CoV2 or HI SARS-CoV2 at 144 h post-infection to look at albumin expression. Samples were plated in triplicate for RT-qPCR. For (A) and (D) Values are expressed as mean ± SEM (∗ = p < 0.05; ∗∗∗∗ = p < 0.0001 based on two-tailed unpaired *t* test).

Our single-cell sequencing results showed the induction of IL-6 signaling following SARS-CoV-2 infection of HLOs. However, no increase in IL-6 RNA expression was observed at 48 h post-infection (data not shown). To determine if IL-6 was upregulated at later time points post-infection, we evaluated expression six days post-infection by qPCR, which showed a significant increase in IL-6 expression in HLOs infected with live SARS-CoV-2 compared to HLOs infected with a heat-inactivated virus (Figure 3A). Quantitative analysis of proteins secreted into the media from infected HLOs showed a significant increase in IL-6 secretion as early as 48 h post-infection (Figure 3B). IL-6 secretion remained elevated through six days post-infection in line with the expression results. No increase of secreted IFNα or IFNγ was observed (Figure 3C), consistent with reports on SARS-CoV-2 infection of hPSC-derived lung alveolar type II cells, which showed no significant induction of IFN genes by four days post-infection (Huang et al., 2020).

Multiple studies have shown an association between severe SARS-CoV-2 infection and a reduction in serum albumin levels (Turcato et al., 2021; Xu et al., 2021; Zeng et al., 2021). To determine if this occurred during SARS-CoV-2 infection of HLOs, qPCR was used to measure albumin expression at six days post-infection. Our results showed a significant reduction in albumin expression in SARS-CoV-2 infected HLOs relative to HLOs infected with heat-inactivated virus (Figure 3D), suggesting that SARS-CoV-2 infection of HLOs mimics the pathology observed in patients with respect to reduction in albumin levels.

### Macrophages promote inflammatory pathway signaling in SARS-CoV-2 infected human-induced pluripotent stem cell-derived liver organoids

**Figure 4:**
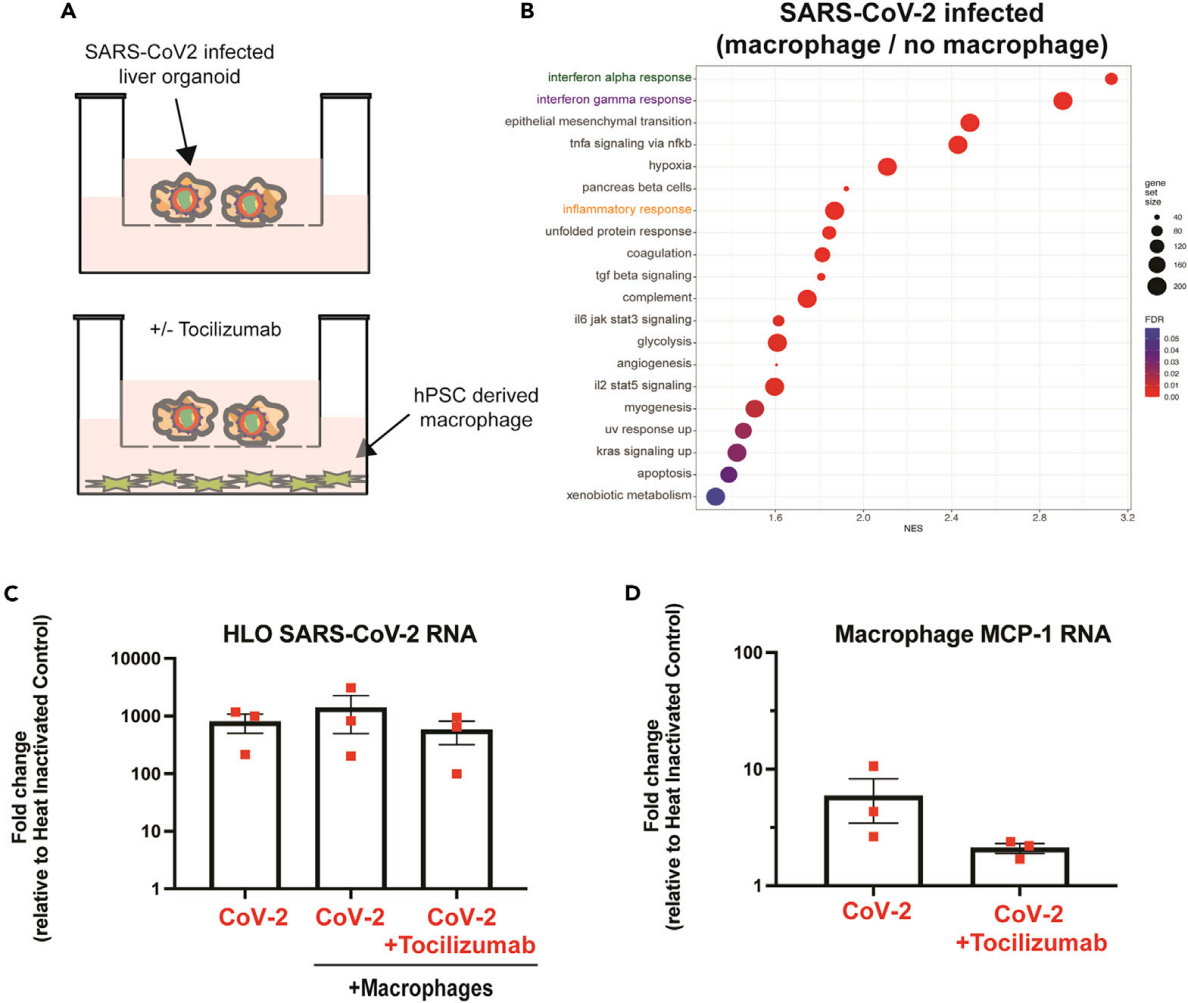
SARS-CoV-2 infected HLOs promote macrophage expression of MCP-1 in an IL-6-dependent manner (A) Experimental schematic of co-culture of SARS-CoV-2 infected HLOs with hPSC-derived macrophages (HMacs). Infected HLOs were incubated with HMacs beginning at 48 h post-infection in the presence or absence of Tocilizumab (5 μg/mL). (B) Gene set enrichment analysis (GSEA) showing the top upregulated and gene sets in infected HLO cultured with HMacs versus infected HLOs not cultured with HMacs. (C) RT-qPCR of SARS-CoV-2 nucleocapsid (NC) expression in HLOs and HMacs 96 h after the initiation of co-culture. (D) RT-qPCR of Monocyte chemoattractant protein-1 (MCP-1) expression in HMacs 96 h after the initiation of co-culture with infected HLOs. All samples were plated in triplicate for RT-qPCR.

IL-6 is known to have proinflammatory effects during viral infection, activating multiple immune cell types, including macrophages (Otsuka and Seino, 2020). To determine if macrophages could impact the transcriptional response of HLOs during infection, we compared gene expression patterns in infected HLOs with and without macrophage exposure. Infected HLOs were placed in the top chamber of a transwell system with hPSC-derived macrophages (HMacs) plated in the lower chamber (Figure 4A). We included the IL-6 receptor blocking antibody tocilizumab in the experiment to evaluate the effects of inhibiting IL-6 signaling. Culturing SARS-CoV-2 infected HLOs with HMacs increased the expression of multiple inflammatory pathways in HLOs, including IFNα and IFNγ, relative to infected HLOs that were not cultured with HMacs (Figures 4B, S7A, and S7B). In addition, exposure to HMacs during infection increased the expression of multiple genes associated with apoptosis (Figures 4B and S7). Incubation of SARS-CoV-2 infected HLOs with both HMacs and tocilizumab did not significantly alter the levels of viral RNA expression in HLOs, suggesting that IL-6 signaling does not restrict viral replication (Figure 4C). The addition of tocilizumab reduced the macrophage-induced upregulation of IFNα and IFNγ signaling; however, there was considerable variability between replicates, suggesting that additional pathways likely contribute to the effects of macrophages on HLOs (Figure S8).

SARS-CoV-2 infection could spread from infected HLOs to HMacs, although viral RNA levels remained higher in HLOs compared to HMacs (Figure S9A). In separate experiments, HMacs were directly infected with SARS-CoV-2. Our results showed a low level of infectious virus production at 24 h post-infection followed by decreased infectious virus production at 48 h post-infection (Figure S9B). These results suggest that although HMacs may be initially susceptible to SARS-CoV-2 infection, unlike HLOs, they do not support sustained infection by SARS-CoV-2. This data is consistent with infection primary monocytes which showed aborted virus production by 48 h post-infection (Junqueira et al., 2022).

To determine if HLO secretion of IL-6 could promote macrophage activation we investigated the levels of Monocyte chemoattractant protein-1 (MCP-1) in HMacs following incubation with infected HLOs. MCP-1 has been shown to regulate the migration and infiltration of monocytes/macrophages (Deshmane et al., 2009). Co-culture of HMacs with infected HLOs increased HMac expression of MCP-1 (Figure 4D). Because IL-6 has been shown to promote MCP-1 expression, we next examined if blocking IL-6 signaling would reduce SARS-CoV-2-mediated induction of MCP-1 expression (Biswas et al., 1998; Tieu et al., 2009). Blocking IL-6 signaling during the incubation of HLOs with HMacs reduced the observed increase in MCP-1 expression (Figure 4D), in spite of robust expression of IL-6 in infected HLOs (Figure S9C). This result indicates that IL-6 signaling upregulates macrophage MCP-1 expression following exposure to SARS-CoV-2-infected HLOs.

## Discussion

The SARS-CoV-2 pandemic has impacted millions of people worldwide. Although primarily a respiratory disease, multiple organ systems can be infected and exhibit deleterious phenotypes. In this report, we present a model to study SARS-CoV-2 infection of human tissues by generating liver organoids from pluripotent stem cells. Our data indicate that SARS-CoV-2 replicates in liver cells leading to rapid changes in gene expression. The most affected gene sets are inflammatory signaling pathways, including interferon and IL-6 signaling. Within the HLOs, hepatocyte-like cells represent the cell type with the strongest induction of inflammatory signaling upon SARS-CoV-2 infection. This result suggests that hepatocytes might be the primary source of inflammation during SARS-CoV-2 infection of the liver. Consistent with this hypothesis, sequencing of liver tissue from SARS-CoV-2-infected patients showed that hepatocytes expressed higher levels of several inflammatory genes relative to cholangiocytes. Our results also show that there is significant induction of inflammatory signaling in cells that have no detectable infection but reside in HLOs exposed to live virus. Although it is possible that these cells were infected at a level below the limit of detection of single-cell sequencing, this result may also indicate that uninfected "bystander" cells contribute to the overall inflammation observed during infection. The significant contribution of uninfected bystander cells to the inflammatory response during SARS-CoV-2 infection has been previously observed in infection stem cell derived intestinal organoids (Triana et al., 2021). Our bulk RNA sequencing results showed that at early time points post-infection the viral particles themselves may be the primary driver of inflammatory signaling in the cells, as both live virus and heat-inactivated virus generated similar responses. However, viral replication is necessary to sustain and amplify this response, as evidenced by our gene expression data of HLOs five days after viral exposure. This result may also reflect the high number of non-infectious “defective” particles that are generated by most viruses (Sanjuan, 2018). The ratio non-infectious to infectious viral particles has been estimated at anywhere from 500 to 10,000 for most animal viruses (Sanjuan, 2018). For this reason, it is possible that even in the samples exposed to live virus only a small fraction of the cells within the HLO are exposed to infectious viral particles.

Our observation showing the activation of interferon signaling cascades in both SARS-CoV-2 infected cells as well as uninfected bystander cells contrasts with a recent report on SARS-CoV-2 infection of intestinal organoids, which showed that interferon signaling was restricted to uninfected bystander cells (Triana et al., 2021). It is possible that, in contrast to the intestine, the liver is a unique environment in which SARS-CoV-2 cannot effectively dampen the interferon response. Alternatively, differences in the threshold for defining a cell as infected may account for some of these discrepancies. In our analysis, we defined an infected cell as having more than one read mapped to the viral genome. This method presumably allows for the detection of cells at the very early stages of infection prior to the generation of sufficient levels of the viral proteins responsible for dampening the cells' innate immune response (Hayn et al., 2021). This hypothesis is consistent with our observation of decreased expression of several IFN-induced genes in cells with greater than ten reads mapped to the viral genome (Figure 2F).

It has been hypothesized that macrophages could promote liver damage following SARS-CoV-2 infection (Licata et al., 2021). Our results showed that co-culture of infected HLOs with HMacs during increased expression of genes associated with apoptosis and inflammatory signaling. We hypothesized that IL-6 signaling could be a mechanism that alerts the immune system to infected liver tissue and promotes the infiltration of macrophages, which has been observed in liver samples from SARS-CoV-2 patients (Fiel et al., 2021; Xu et al., 2020). We also observed sustained IL-6 secretion in infected HLOs. To test this hypothesis, we co-cultured macrophages with infected HLOs without direct physical contact. Co-culture with infected HLOs potently upregulated macrophage MCP-1, which is at least in part dependent on IL-6 signaling, as tocilizumab blunted MCP-1 expression. Additionally, this reduced inflammatory gene expression in the HLOs, showing a clear crosstalk between HLOs and macrophages. Several clinical trials provide evidence that tocilizumab may reduce mortality in certain patients with COVID-19. Our study recapitulates a mechanism for this phenomenon *in vitro*, showing that IL-6 receptor blocking can dampen a potentially harmful inflammatory response.

In conclusion, this report is a proof-of-concept that modeling SARS-CoV-2 infection with hPSC-derived tissues is possible. It should be noted that stem cell-derived HLOs likely contain cells at earlier stages of differentiation than what would be typically found in the adult liver, therefore, may not fully represent the response to infection. However, the isogenic nature of the cells and the ease with which this system can be scaled to meet the needs of rigorous experimentation perfectly sets up these cell models as tools to investigate inflammatory mechanisms and test the efficacy of drugs proposed to improve patient recovery after COVID-19 infection.

### Limitations of the study

This study focuses on the infection of hPSC-derived liver organoids with the original SARS-CoV-2 strain. Although HLOs recapitulate multiple cell types present in the liver, they do not perfectly recapitulate the *in vivo* liver in terms of maturity and cell type diversity. The re-aggregation and infection process may further stress the cells to dedifferentiate owing to damage. The viral strain used here does not contain many of the mutations that are present in the current predominant infectious strain.

## STAR★Methods

### Key resources table

**Table:** 

REAGENT or RESOURCE	SOURCE	IDENTIFIER
**Antibodies**		

Sars-CoV-2 Nucleocapsid	GeneTex	GTX135357;RRID: AB_2868464
HNF4a	Life Technologies	MA1-199;RRID: AB_2633309
dsRNA	SCICONS English & Scientific Consulting	J2;RRID: AB_2651015
KRT17	Abcam	ab53707;RRID: AB_869865

**Bacterial and virus strains**		

SARS-CoV-2 (USA_WA1/2020)	BEI	NR-52281

**Chemicals, peptides, and recombinant proteins**		

Tocilizumab	Selleck	A2012

**Critical commercial assays**		

RNeasy Micro Kit	Qiagen	74004
Chromium Next GEM Automated Single Cell 3′ Library and Gel Bead Kit v3.1	10X Genomics	1000147
Custom Luminex Assay	R&D Systems	NA

**Deposited data**		

Raw and analyzed sequencing data	This study	GEO: GSE210061

**Experimental models: Cell lines**		

Vero E6	ATCC	CRL-1586
H1 (WA01) hPSCs	WiCell	NA
Allen hPSCS	the Allen Institute	NA
1016 (DiPS 1016 SevA) hPSCs	Harvard Stem Cell Institute iPS Core	NA

**Oligonucleotides**		

Primers for RPLP0, SARS-CoV-2, Albumin, HNF4a, IL-6, MCP-1 See Table S3	N/A	N/A

**Software and algorithms**		

Code used to create Figures 2A and S3	This study	Code to create Figures 2A and S3.zip

### Resource availability

#### Lead contact

Further information and requests for resources and reagents should be directed to and will be fulfilled by the lead contact, Dr. Rudolf Jaenisch (jaenisch@wi.mit.edu).

#### Materials availability

This study did not generate new unique reagents.

### Experimental model and subject details

#### hPSC lines and maintenance

hPSC lines used in this study were obtained from WiCell (H1), the Allen Institute (Allen line), the Harvard Stem Cell Institute iPS Core (DiPS 1016 SevA). hPSC lines were maintained in feeder-free conditions in StemFlex (Gibco) media on Matrigel (Corning) in 6-well tissue culture dishes. For passaging, hPSCs were detached as clumps using Versene Solution (Thermo Fisher) and replated at a ratio of 1:8–1:10.

#### Directed differentiation of hPSCs to HLOs

The HLO differentiation protocol is adapted from a previously published protocol (Ouchi et al., 2019). Briefly, hPSCs were detached and seeded on Matrigel coated tissue culture plates. Medium was changed to RPMI 1640 medium containing 100 ng/mL Activin A and 50 ng/mL bone morphogenetic protein 4 (BMP4) on day 1, 100 ng/mL Activin A and 0.2% fetal bovine serum (FBS) on day 2, and 100 ng/mL Activin A and 2% FBS on day 3. On day 4–8, cells were cultured in Advanced DMEM/F12 with 500 ng/mL fibroblast growth factor 4 and 3uM CHIR99021. On day 9, cells were gently pipetted to delaminate from the dish, centrifuged at 800rpm for 3 minutes and resuspended at 1 × 10^6^ cells/mL in Advanced DMEM/F12 with 2uM retinoic acid and plated into ultra-low attachment 6 well plates on an orbital shaker at 95rpm and fed daily. On day 14 the medium was switched to Hepatocyte Culture Medium (HCM, Lonza) with 10 ng/mL hepatocyte growth factor, 0.1uM dexamethasone and 20 ng/mL Oncostatin M. Cultures were fed daily until used for assays.

#### SARS-CoV-2 propagation, titration and inactivation

SARS-CoV-2 (isolate USA_WA1/2020) was obtained from BEI Resources. The virus was propagated in Vero E6 cells (ATCC CRL-1586) cultured in Dulbecco’s modified Eagle’s medium (DMEM) supplemented with 2% fetal calf serum (FCS), penicillin (50 U/mL), and streptomycin (50 mg/mL). SARS-CoV-2 titer was determined in Vero E6 cells by plaque assay. All work with SARS-CoV-2 was performed in the biosafety level 3 (BSL3) at the Ragon Institute (Cambridge, MA) following approved SOPs. To generate heat-inactivated SARS-CoV-2 a 1mL aliquot of SARS-CoV-2 was heated to 85°C for 15 minutes.

#### SARS-CoV-2 infection of HLOs

At approximately day 25 of differentiation HLOs were incubated in 0.25% trypsin for 30 minutes followed by mechanical dissociation by repeated pipetting using a p200 pipette. HLO organoid fragments were resuspended in re-aggregation media (HCM +10% FBS and 4uM y-27632) and approximate cell number determined. HLO fragments were incubated with SARS-CoV-2 at a multiplicity of infection of 0.3 for 1 h at 37C and 5% CO2. For mock infections HLO fragments were incubated with Vero cell conditioned media (DMEM with 2% FBS). After the adsorption period, HLO fragments were pelleted by low-speed centrifugation (500g x5 min) and resuspended in re-aggregation media. At 24hr post-infection re-aggregation media was replaced with HLO media for the duration of the infection.

#### Directed differentiation of hPSCs to HMacs

HMacs were derived from the H1 iPSC line. Myeloid progenitor cells were derived from H1 embryonic stem cells (ESC) as previously described (Brownjohn, 2018). Briefly, H1 ESCs were cultured and maintained in feeder-free culture conditions in StemFlex (Gibco) media. At approximately 70% confluence, the medium was transitioned to Essential 8 (ThermoFisher Scientific) pluripotent stem cell medium supplemented with P/S and 10 μM Y-27632 (ROCK) inhibitor (Stem Cell Technologies) on day −1. On day 0, the H1 ESCs were dissociated into a single cell suspension using TrypLE Express (ThermoFisher Scientific) and diluted to 0.666 × 10^6^ per mL in embryoid body (EB) medium consisting of complete E8 medium with P/S supplemented 50 ng/mL bone morphogenetic protein 4 (Peprotech), 20 mg/mL stem cell factor (Peprotech), and 50 ng/mL vascular endothelial growth factor, with 10 μM ROCK inhibitor. 150 μL of this cell suspension per well was plated into a 96-well U-bottom ultra-low adherence well plates (Corning) and centrifuged at 200 RCF for 5 min. On day 2, 150 μL of EB medium without ROCK inhibitor was added to each well. On day 4, the EBs were transferred to a 10 cm tissue culture-treated polystyrene dish in 12 mL of hematopoetic myeloid (HpM) medium consisting of X-VIVO 15 medium (Lonza) supplemented with 2 mM GlutaMax (Gibco), 55 μM beta-mercaptoethanol (Sigma Aldrich), 100 ng/mL macrophage colony-stimulating factor (Peprotech), and 25 ng/mL interleukin-3 (Peprotech). Media was replaced with fresh HM media every 3–4 days. At 2–3 weeks after EB plating, floating CD14^+^ myeloid precursors (MPs) were collected and differentiated into macrophages by plating the cells in a 10 cm tissue culture-treated polystyrene dish in 12 mL serum-free macrophage medium (SFMM) consisting of glutamine containing no glucose RPMI medium (Gibco) supplemented with 1 mM glucose (Sigma Aldrich), 0.432 mg/L zinc sulfate heptahydrate (Sigma Aldrich), 7.5 mg/L human transferrin (holo) (Sigma Aldrich), 1.9 mg/L ethanolamine (Sigma Aldrich), 0.005 mg/L sodium selenite (Sigma Aldrich), 1.7 nM human insulin (Sigma Aldrich), 1:100 dilution of minimal essential amino acids (Gibco), 1:100 dilution of sodium pyruvate (Gibco), P/S, 0.2 g/L of recombinant human albumin (CellaStim), and 100 ng/mL macrophage colony-stimulating factor. The MPs were differentiated into macrophages over 10 days in SFFM with the same feeding schedule as the EBs in HpM medium.

#### HLO co-culture with hPSC derived macrophages

HLOs were infected with SARS-CoV-2 as previously described. At 48 hours post infection infected HLOs and the associated media was transferred to the insert of the 12-well transwell plate (Corning # 3460). HMacs were plated in 12 well plates with approximately 1 × 10 ^5^ cells plated per well. Transwell inserts containing HLOs and associated media were transferred to HMac containing 12 well plates for 96 hours at which point HLOs and HMacs were collected and cellular RNA isolated.

### Method details

#### Immunofluorescence microscopy of HLOs

Infected or control HLOs were fixed in 4% PFA for 30min, washed twice in PBS, then incubated in 30% sucrose at 4°C for 48 hours. Sucrose was then removed and replaced with OCT and samples incubated at 4°C overnight. Samples were then frozen in OCT at −20°C overnight. Samples were then cut into 10μm sections and mounted on glass slides and stored at −20°C. At the time of staining slides were equilibrated to room temperature for 20 minutes and then washed three times in PBS. Slides were blocked for two hours in PBS w/0.1% TX-100, 10% normal goat serum (NGS). Slides were then incubated with primary antibodies (see table below) diluted in PBS w/0.1%TX-100, 1% BSA, 1% NGS overnight at 4°C. Slides were then washed in PBST and incubated with secondary antibodies and DAPI diluted in PBS w/0.1%TX-100, 1% BSA, 1% NGS for 1 hour at room temperature. After washing with PBST glass coverslips were mounted on sections using ProLong Diamond Antifade Mountant (ThermoFisher).

#### RNA isolation, cDNA preparation, and qRT-PCR

RNA from HLOs was extracted using the RNeasy Plus Mini kit (QIAGEN) following the manufacturer’s protocol. cDNA synthesis was performed using qScript cDNA Supermix according to the manufacturer’s instructions, using 1000ng RNA as input (qScript cDNA SuperMix, QuantaBio - 95048-500). qPCR was performed on a Thermo Fisher Scientific QuantStudio 6 machine using Fast SYBR™ Green Master Mix (Thermo Fisher Scientific 4385618). Expression data is presented after calculating the relative expression compared with the housekeeping gene RPLP0, using the equation Relative Quantification (RQ) = 100/(2ˆ(Target Gene Ct – RPLP0 Ct). When data is reported relative to a sample condition, the condition of reference was set as 1 and the data of the other conditions were reported as a ratio (condition/condition of reference).

#### Single-cell RNA-sequencing

HLOs were collected and dissociated into single cell suspensions using 0.25% trypsin. Single-cell suspensions were loaded onto the Chromium Controller (10x Genomics) for droplet formation. scRNA-seq libraries were prepared using the Chromium Single Cell 3′ Reagent Kit (10x Genomics). Samples were sequenced using a HiSeq 2500 system. Fastq files were generated with cellranger-4.0.0 mkfastq. We used “cellranger-4.0.0 mkgtf” to make a gtf file containing human protein coding genes, antisense genes, lincRNAs as well as exon coding for the TCR and BRC, as recommended by 10x genomics. We added annotations for viral genes and the 3 prime viral UTR to the gtf file. To be able to detect where on the negative strand viral reads were mapping we also added annotation for viral genes where the strand was set to “-.” We created a reference genome with “cellranger-4.0.0 mkref” using as input the gtf file described above and a fasta file containing the human genome sequence from ENSEMBL release 93 (ftp://ftp.ensembl.org/pub/release-93/fasta/homo_sapiens/dna/Homo_sapiens.GRCh38.dna.primary_assembly.fa.gz) concatenated to the SARS-CoV-2 sequence, GenBank ID: MN988713.1. Read mapping and assigning reads to genes was done with “cellranger-4.0.0 count.” We analyzed the scRNA-seq data with the R package Seurat 4.0.3 (Butler, 2018). We integrated the two samples using the canonical correlation analysis (CCA) implemented on the Seurat functions FindIntegrationAnchors and IntegrateData, following the tutorial “https://satijalab.org/seurat/archive/v3.0/immune_alignment.html.” We used the integrated assay to run PCA and UMAP and find clusters using at a resolution of 0.05. This clustering strategy resulted on four clusters. Based on hepatocyte and cholangiocyte specific marker expression we labelled clusters 0,and 3 as hepatocyte-like cells-1, cluster 1 as hepatocyte-like cells-2, and cluster 2 as cholangiocyte like cells. We used the RNA assay to make the dotplots as well as the figures showing expression of individual genes on the UMAP visualizations. For differential expression analysis and to make the dotplots figures we removed cells that had less than 200 genes detected or more than 20% of the reads mapping to mitochondrial genes. We classified cells as infected, where we detect the virus, if they contained more than one read mapping to the positive strand of the viral genome. There were 12 cells with more than one read mapping to the viral genome on the samples treated with heat inactivated virus (Table S1). These reads may come from the input virus or from unproper mapping. We removed those cells prior to performing the differential expression analysis and making the dotplot figures. To find genes differentially expressed on the scRNA-seq data, we extracted, from the Seurat object, the sum of the counts of all the cells in the condition and sample to be compared; *i.e*. infected cells (reads mapped to the virus >1), bystandard cells (0 or 1 read mapped to the virus), and the cells treated with HI virus, for each cell line (H1 ESCs or 1016 iPSCs). We then perform DE analysis with DESeq-2 (Love, 2014) with these pseudocounts and including the cell line of origin in the model. That is, we compared the infected cells from each organoid to the cells treated with HI virus from the same organoid of origin. We used the statistic generated by DESeq-2 to rank the genes and perform gene set enrichment analysis (GSEA) (Subramanian, 2005) (using the hallmark gene sets version 7.1 (h.all.v7.1.symbols.gmt) from the Molecular Signatures Database (MSigDB) (Subramanian, 2005; Liberzon, 2015; Liberzon, 2011). We clustered the DE genes visualized on the heatmaps with cluster 3 (Hoon, 2004) and made the heatmaps with a custom R script. For average expression values, each dot color represents the average expression of that gene in the cells included in that condition (cluster1 HI, cluster1 CoV-2 etc). For each gene the average expression is scaled across all conditions ( the mean for that gene across conditions is subtracted from the average of the cells in each condition/dot), and then is divided by the standard deviation.

#### Integration of the scRNA-seq from organoids with the snRNA-seq data from liver autopsies

The sn-RNA-seq liver autopsy samples and cell identity annotations were downloaded from the Broad single cell portal. To create a Seurat object, we followed the vignette “https://mojaveazure.github.io/seurat-disk/articles/convert-anndata.html”. Briefly, we loaded the SeuratObject and SeuratDisk libraries and used the “Convert” function from “SeuratDisk” to covert and save the h5ad file a Seurat object. The saved 'Seurat' object was loaded using “LoadH5Seurat”. To integrate the liver autopsy samples and our integrated sc-RNA-seq from organoid samples, we first found the genes common in both datasets, then extracted Seurat objects containing the only common genes, and finally integrated the two objects using “FindIntegrationAnchors” and “IntegrateData” Seurat functions. The cell annotation used on the liver autopsy samples was the predicted annotation provided by the Broad Institute. One of the cell annotations was “b”; we relabeled those as “B cells”. To integrate only the hepatocytes and cholangiocytes from the liver autopsies with our data, we first subset the Seurat object containing the autopsy samples and extracted the cells labeled as the hepatocytes and cholangiocytes, on the predicted annotation; we then integrated those with our integrated organoid samples with Seurat as described above.

#### Bulk RNA-sequencing of HLOs

For bulk RNA sequencing of total RNA libraries were prepared using the Swift RNA library prep kit (Swift Biosciences). Samples were sequenced on a NovaSeq 6000 sequencer.

#### Analysis of bilk RNA-sequencing data

Fifty one nt long paired end reads were mapped with STAR (Dobin, 2013) limiting the intron length (--alignIntronMax) to 95000. We used human genome version hg38 with ENSEMBL annotation GRCh38.93. We added the SARS-CoV2 sequence (GenBank ID: MN988713.1) and annotation to the genome fasta and gtf files before making a genome index. We assigned reads to genes using featureCounts (Liao, 2014) with options “-p -s 2” and the same gtf file used for mapping. Differential expression analysis was done with DESeq2 (Love, 2014) using normal shrinkage. Preranked GSEA (Subramanian, 2005) was on performed on the list of genes ranked by the DESeq2 statistic. Clustering on gene expression was done with Cluster 3 (Hoon, 2004) and heatmaps were drawn with the heatmap R function. We removed genes that had less than one count per sample on average. When the mock samples were included on the heatmap, the normalized counts were divided by the mean of the mock samples and we removed genes with mean 0 in the mock samples. For heatmaps that don’t include mock samples we mean centered the normalized counts.

### Quantification and statistical analysis

Prism software from Graphpad Inc was used for all statistical analyses not related to sequencing results. Comparative analysis was preformed by unpaired t-test. Statistical details of experiments can be found in the figure legends.

## Data Availability

Single-cell and bulk RNA-seq data have been deposited at GEO and are publicly available as of the date of publication. Accession numbers are listed in the key resources table. Microscopy data reported in this paper will be shared by the lead contact upon request. The original code used produce Figures 2A and S3 is provided in the supplementary material (Data S1.zip). Any additional information required to reanalyze the data reported in this paper is available from the lead contact upon request.

## Supplemental information

Document S1. Figures S1–S9

Table S1. Cell counts of populations identified based on reads mapped to the SARSCoV-2 genome, related to Figure 2B

Table S2. Cell counts for clusters identified in HLOs exposed to live SARS-CoV-2, related to Figures 2B and 2E

Table S3. Sequences for primers used in this study, related to Figures 1, 3, 4, and S9

Data S1. Code used to create Figures 2A and S3
