# Supplementary material for: SARS-CoV-2 infection of human pluripotent stem cell-derived liver organoids reveals potential mechanisms of liver pathology
Source: iScience. 2022 Sep 16;25(10):105146. doi: 10.1016/j.isci.2022.105146 (PMC9477603; doi:10.1016/j.isci.2022.105146)
Supplement: Document S1. Figures S1–S9 [file mmc1.pdf]

iScience, Volume *25*

Supplemental information

SARS-CoV-2 infection of human pluripotent stem cell-derived liver organoids reveals potential mechanisms of liver pathology

Alexsia Richards, Max Friesen, Andrew Khalil, M. Inmaculada Barrasa, Lee Gehrke, and Rudolf Jaenisch

Figure S1

(A)

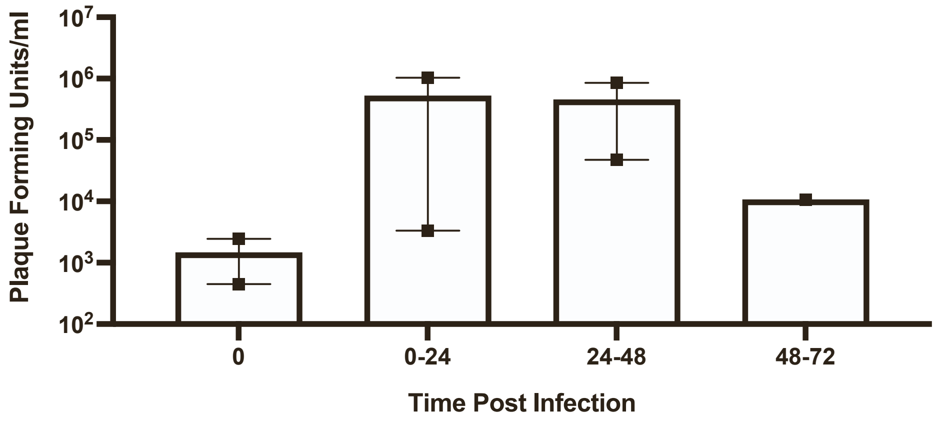

Plaque Forming Units/ml
10^7
10^6
10^5
10^4
10^3
10^2
0
0-24
24-48
48-72
Time Post Infection

(B)

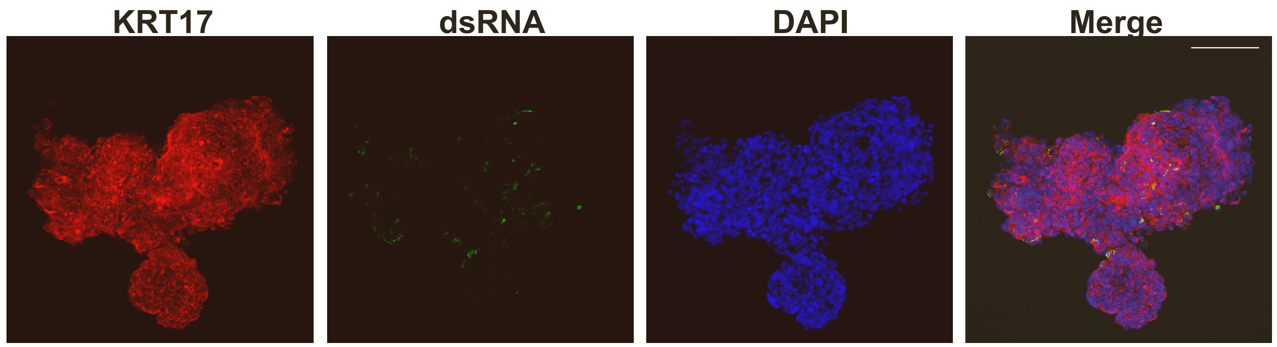

KRT17
dsRNA
DAPI
Merge

Figure S2

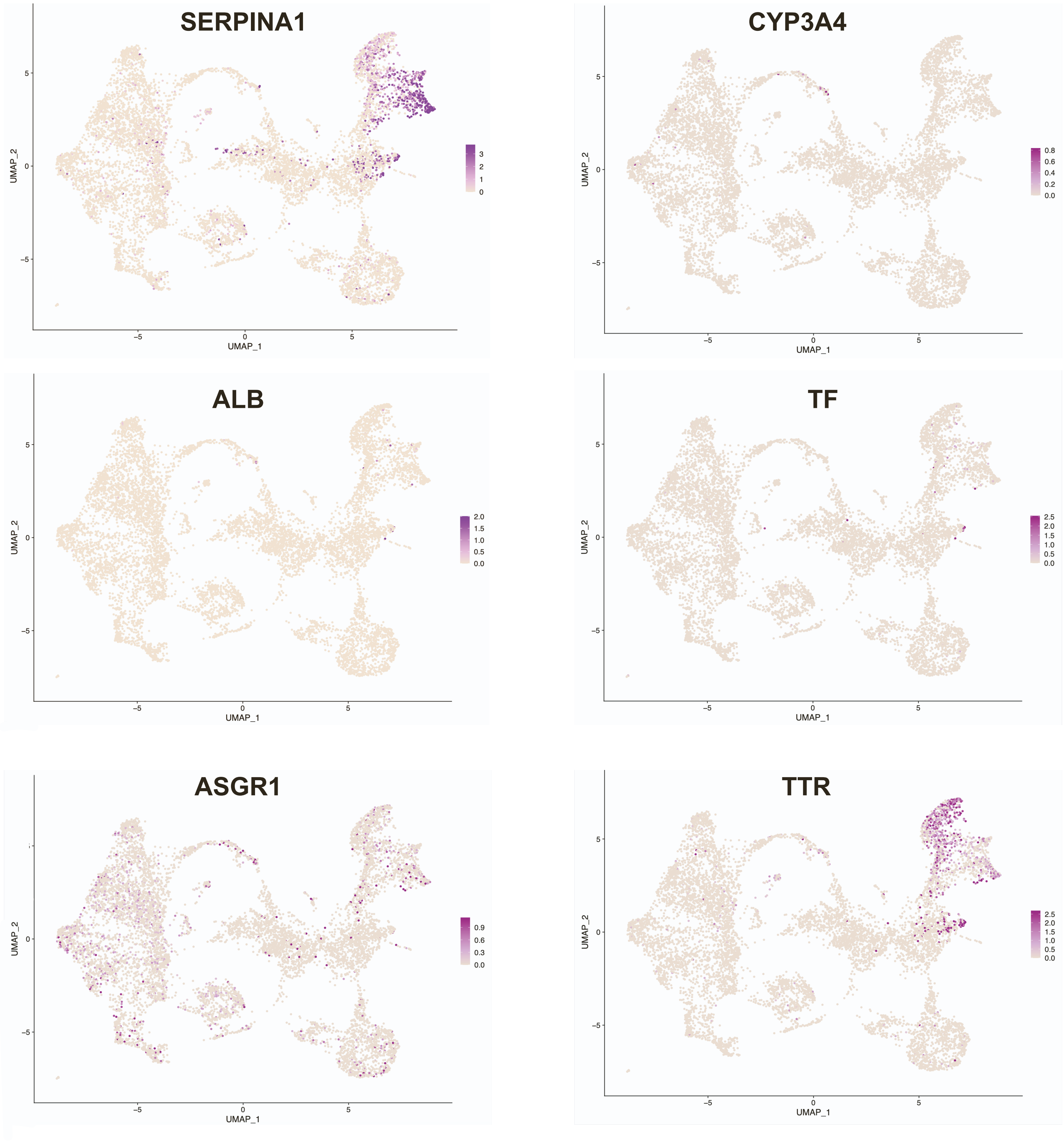

Figure S3

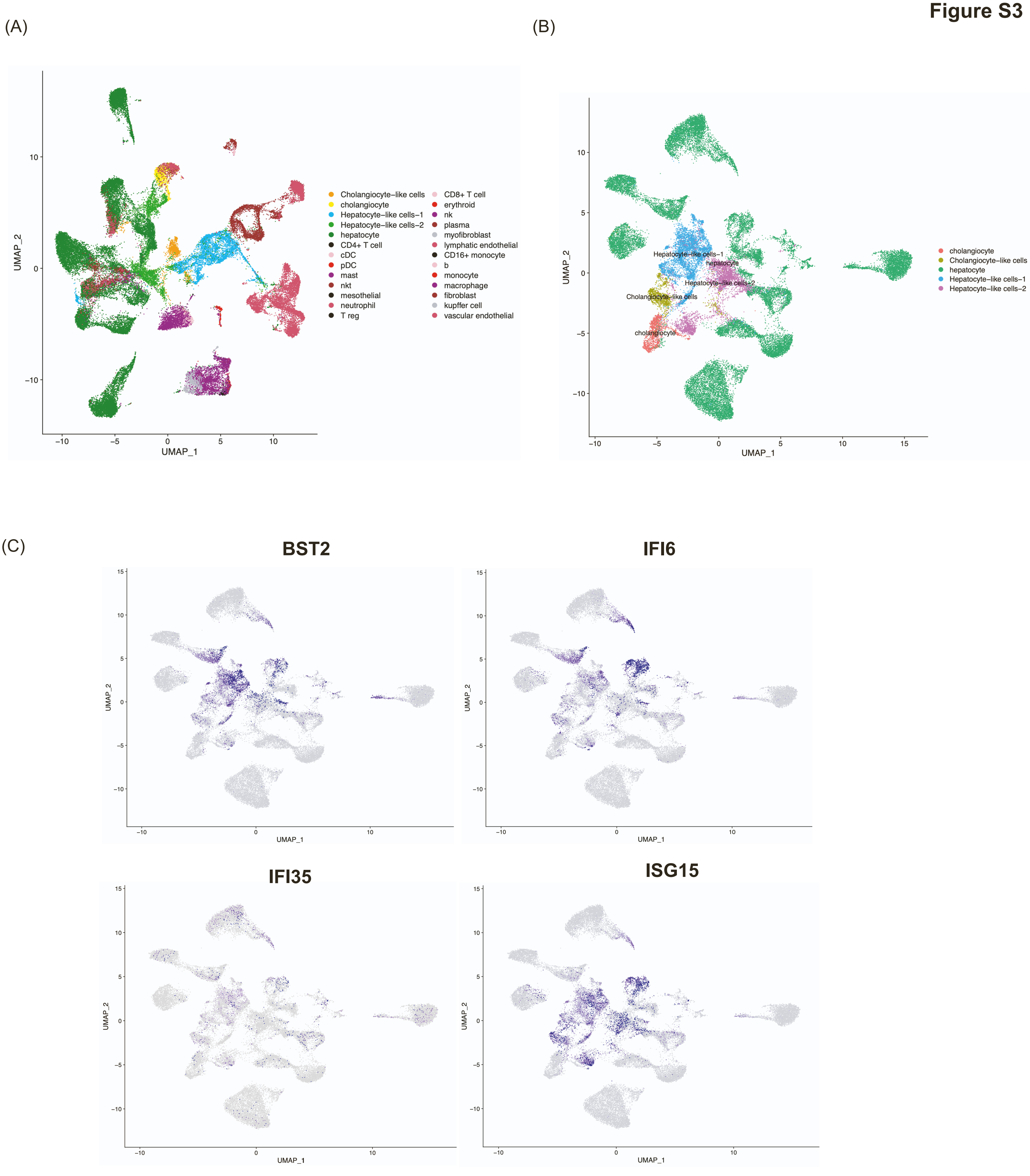

(A)
UMAP_1
UMAP_2
Cholangiocyte−like cells
cholangiocyte
Hepatocyte−like cells−1
Hepatocyte−like cells−2
hepatocyte
CD4+ T cell
cDC
pDC
mast
nkt
mesothelial
neutrophil
T reg
CD8+ T cell
erythroid
nk
plasma
myofibroblast
lymphatic endothelial
CD16+ monocyte
b
monocyte
macrophage
fibroblast
kupffer cell
vascular endothelial
(B)
UMAP_1
UMAP_2
cholangiocyte
Cholangiocyte−like cells
hepatocyte
Hepatocyte−like cells−1
Hepatocyte−like cells−2
(C)
BST2
IFI6
IFI35
ISG15
UMAP_1
UMAP_2

Figure S4

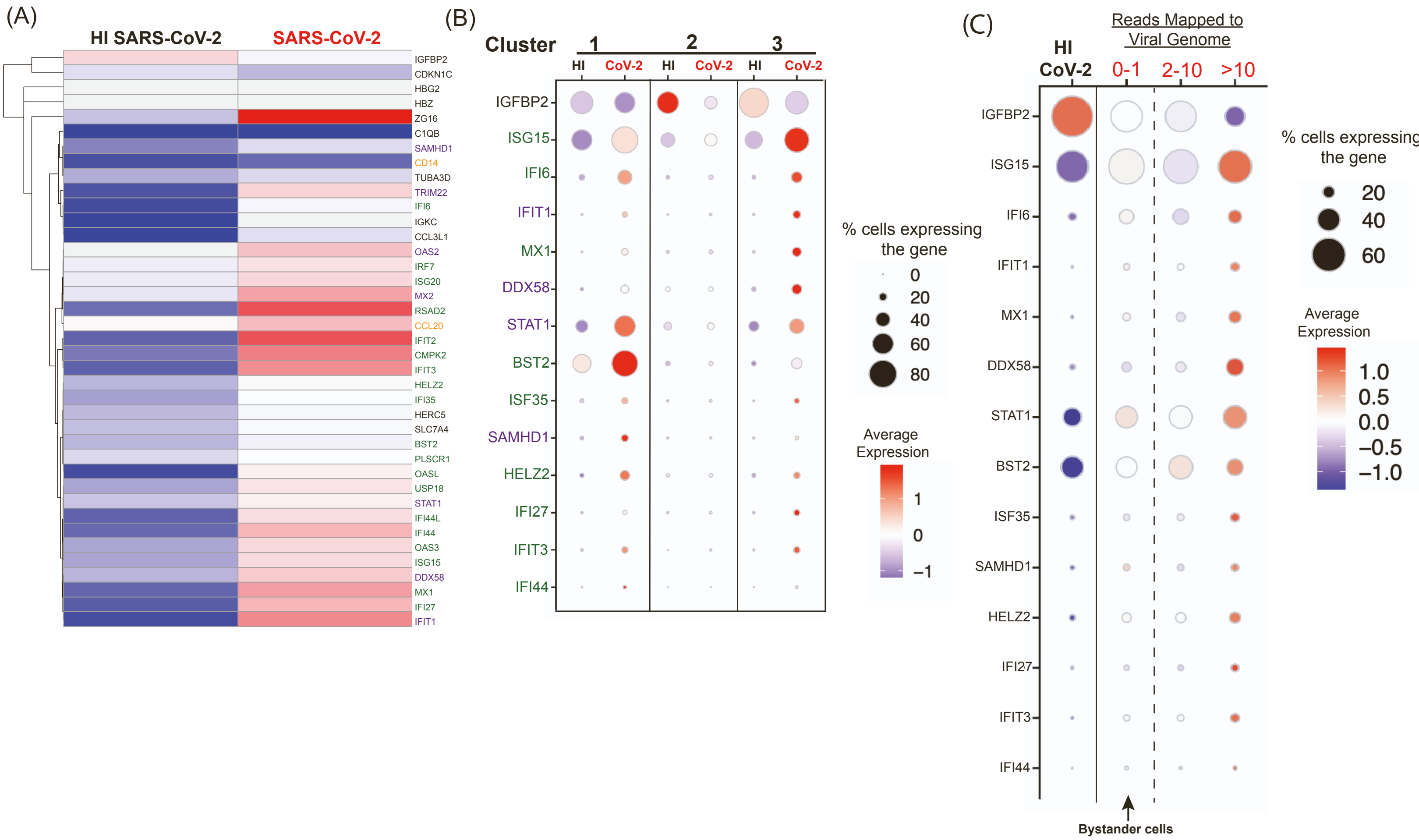

(A)
HI SARS-CoV-2
SARS-CoV-2
IGFBP2
CDKN1C
HBG2
HBZ
ZG16
C1QB
SAMHD1
CD14
TUBA3D
TRIM22
IFI6
IGKC
CCL3L1
OAS2
IRF7
ISG20
MX2
RSAD2
CCL20
IFIT2
CMPK2
IFIT3
HELZ2
IFI35
HERC5
SLC7A4
BST2
PLSCR1
OASL
USP18
STAT1
IFI44L
IFI44
OAS3
ISG15
DDX58
MX1
IFI27
IFIT1
(B)
Cluster
1
2
3
HI
CoV-2
IGFBP2
ISG15
IFI6
IFIT1
MX1
DDX58
STAT1
BST2
ISF35
SAMHD1
HELZ2
IFI27
IFIT3
IFI44
% cells expressing the gene
0
20
40
60
80
Average Expression
1
0
−1
(C)
Reads Mapped to Viral Genome
HI CoV-2
0-1
2-10
>10
IGFBP2
ISG15
IFI6
IFIT1
MX1
DDX58
STAT1
BST2
ISF35
SAMHD1
HELZ2
IFI27
IFIT3
IFI44
Bystander cells
% cells expressing the gene
20
40
60
Average Expression
1.0
0.5
0.0
−0.5
−1.0

Figure S5

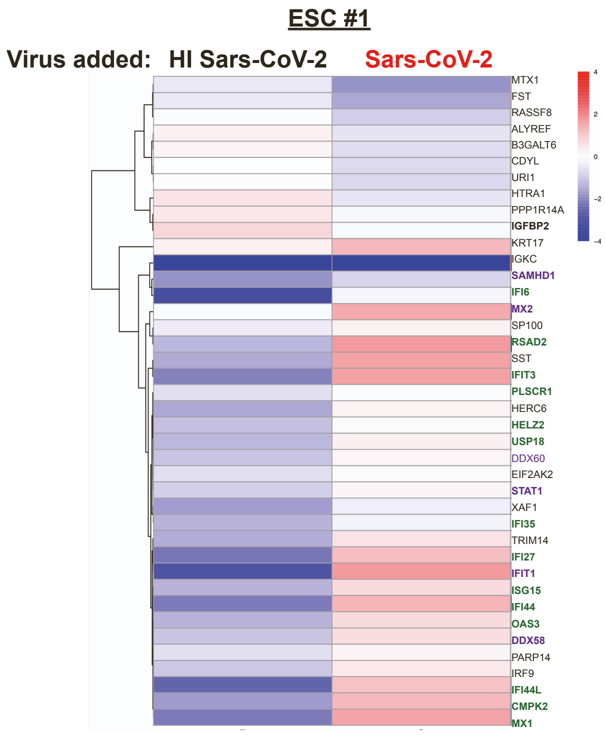
ESC #1
Virus added: HI Sars-CoV-2 Sars-CoV-2
MTX1
FST
RASSF8
ALYREF
B3GALT6
CDYL
URI1
HTRA1
PPP1R14A
IGFBP2
KRT17
IGKC
SAMHD1
IFI6
MX2
SP100
RSAD2
SST
IFIT3
PLSCR1
HERC6
HELZ2
USP18
DDX60
EIF2AK2
STAT1
XAF1
IFI35
TRIM14
IFI27
IFIT1
ISG15
IFI44
OAS3
DDX58
PARP14
IRF9
IFI44L
CMPK2
MX1
4
2
0
−2
−4

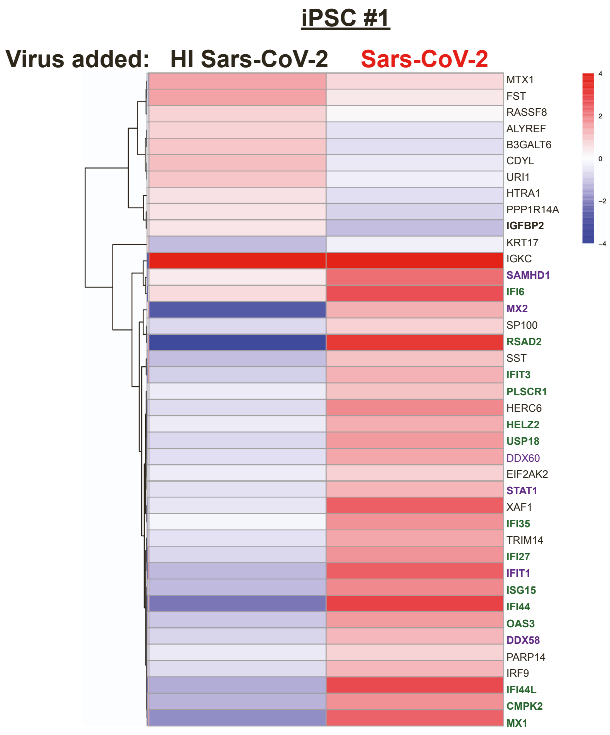
iPSC #1
Virus added: HI Sars-CoV-2 Sars-CoV-2
MTX1
FST
RASSF8
ALYREF
B3GALT6
CDYL
URI1
HTRA1
PPP1R14A
IGFBP2
KRT17
IGKC
SAMHD1
IFI6
MX2
SP100
RSAD2
SST
IFIT3
PLSCR1
HERC6
HELZ2
USP18
DDX60
EIF2AK2
STAT1
XAF1
IFI35
TRIM14
IFI27
IFIT1
ISG15
IFI44
OAS3
DDX58
PARP14
IRF9
IFI44L
CMPK2
MX1
4
2
0
−2
−4

Figure S6

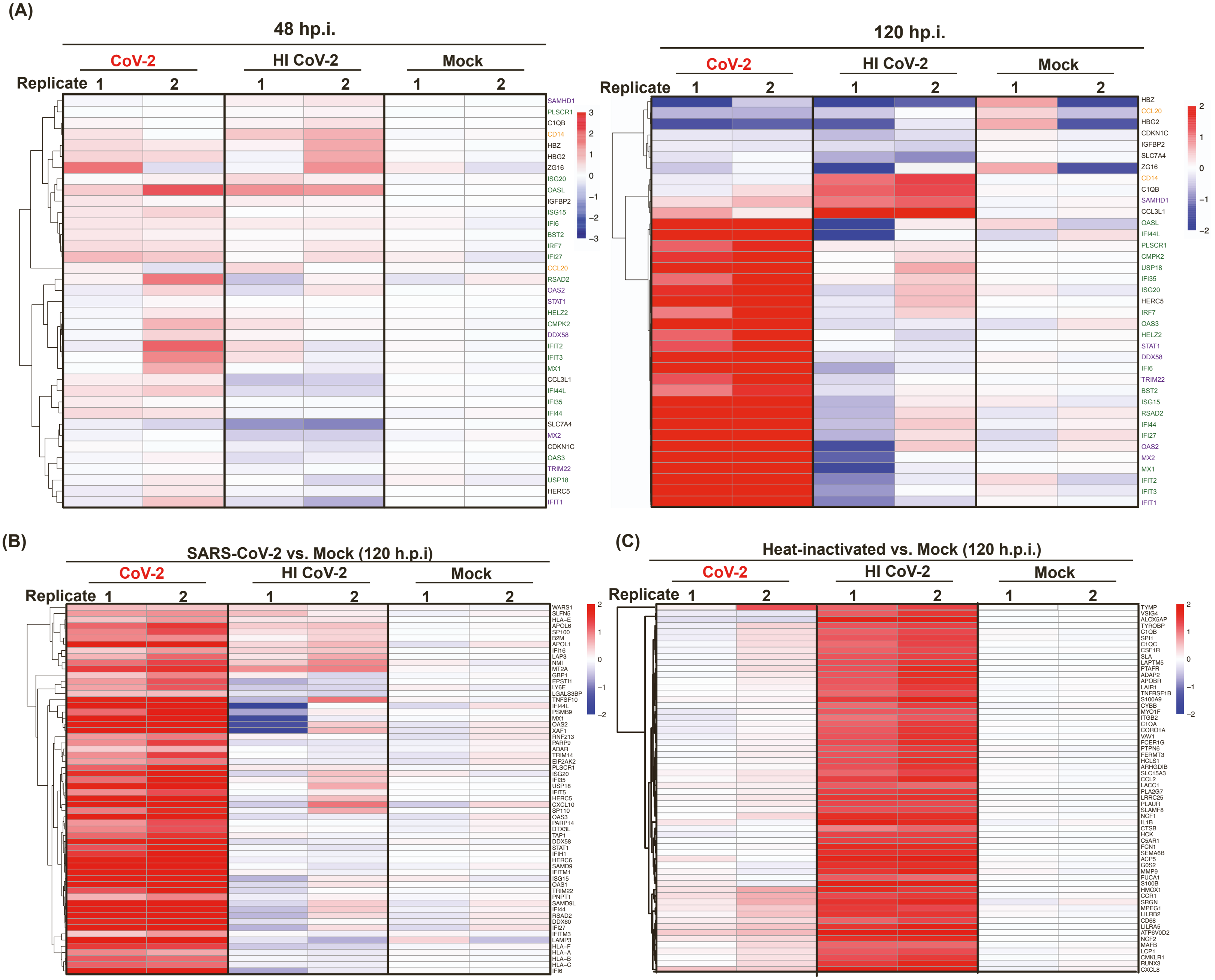
(A)
48 hp.i.
120 hp.i.
CoV-2
HI CoV-2
Mock
Replicate 1 2 1 2 1 2
(B)
SARS-CoV-2 vs. Mock (120 h.p.i)
(C)
Heat-inactivated vs. Mock (120 h.p.i.)

Figure S7

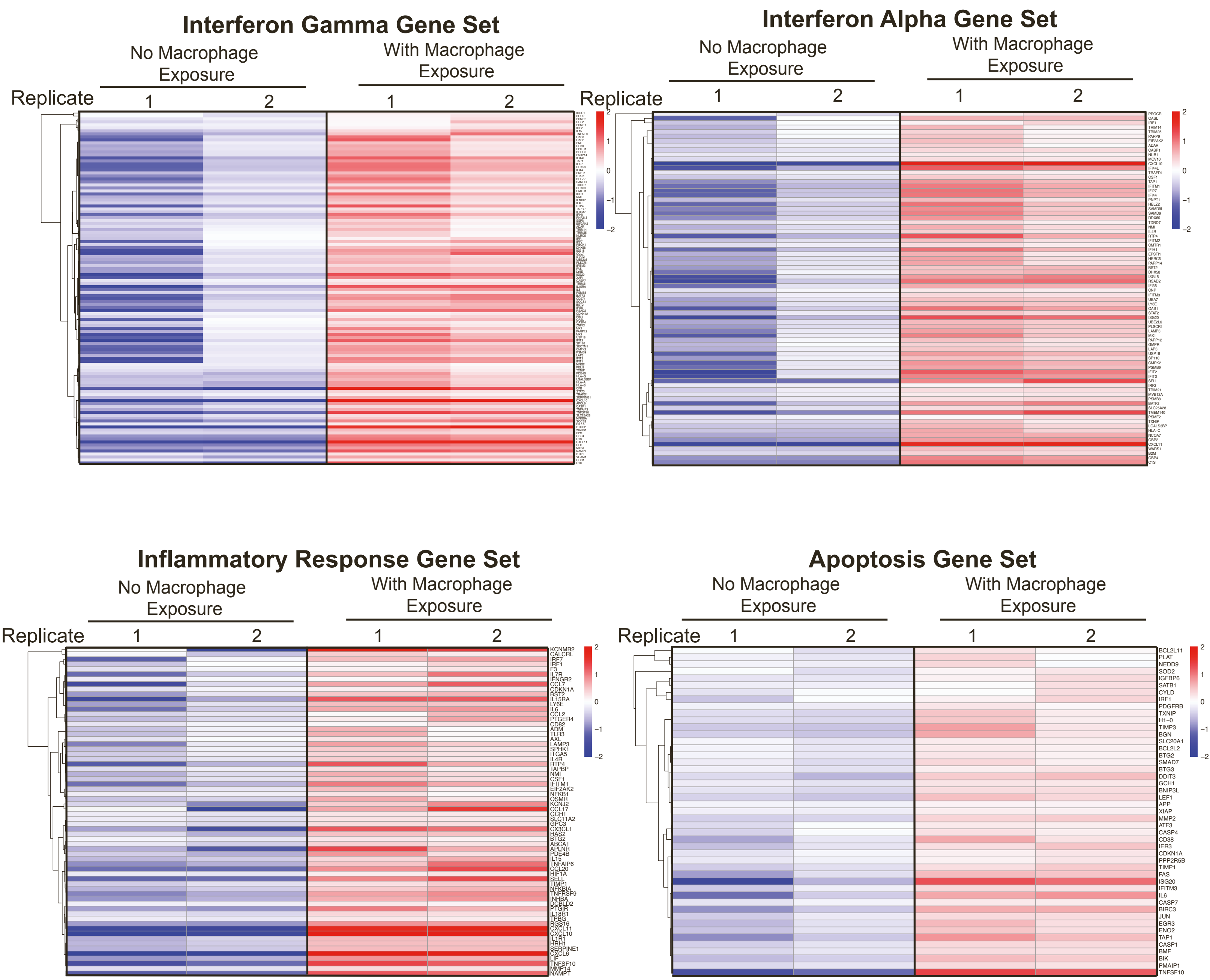
Interferon Gamma Gene Set
No Macrophage Exposure
With Macrophage Exposure
Replicate
1
2
1
2
Interferon Alpha Gene Set
No Macrophage Exposure
With Macrophage Exposure
Replicate
1
2
1
2
Inflammatory Response Gene Set
No Macrophage Exposure
With Macrophage Exposure
Replicate
1
2
1
2
KCNMB2
CALCRL
IRF7
IRF1
F3
IL7R
IFNGR2
CCL7
CDKN1A
BST2
IL15RA
LY6E
IL6
CCL2
PTGER4
CD82
ADM
TLR3
AXL
LAMP3
SPHK1
ITGA5
IL4R
RTP4
TAPBP
NMI
CSF1
IFITM1
EIF2AK2
NFKB1
OSMR
KCNJ2
CCL17
GCH1
SLC11A2
GPC3
CX3CL1
HAS2
BTG2
ABCA1
APLNR
PDE4B
IL15
TNFAIP6
CCL20
HIF1A
SELL
TIMP1
NFKBIA
TNFRSF9
INHBA
DCBLD2
PTGIR
IL18R1
TPBG
RGS16
CXCL11
CXCL10
IL1R1
HRH1
SERPINE1
CXCL6
LIF
TNFSF10
MMP14
NAMPT
Apoptosis Gene Set
No Macrophage Exposure
With Macrophage Exposure
Replicate
1
2
1
2
BCL2L11
PLAT
NEDD9
SOD2
IGFBP6
SATB1
CYLD
IRF1
PDGFRB
TXNIP
H1-0
TIMP3
BGN
SLC20A1
BCL2L2
BTG2
SMAD7
BTG3
DDIT3
GCH1
BNIP3L
LEF1
APP
XIAP
MMP2
ATF3
CASP4
CD38
IER3
CDKN1A
PPP2R5B
TIMP1
FAS
ISG20
IFITM3
IL6
CASP7
BIRC3
JUN
EGR3
ENO2
TAP1
CASP1
BMF
BIK
PMAIP1
TNFSF10
2
1
0
−1
−2

Figure S8

(A)

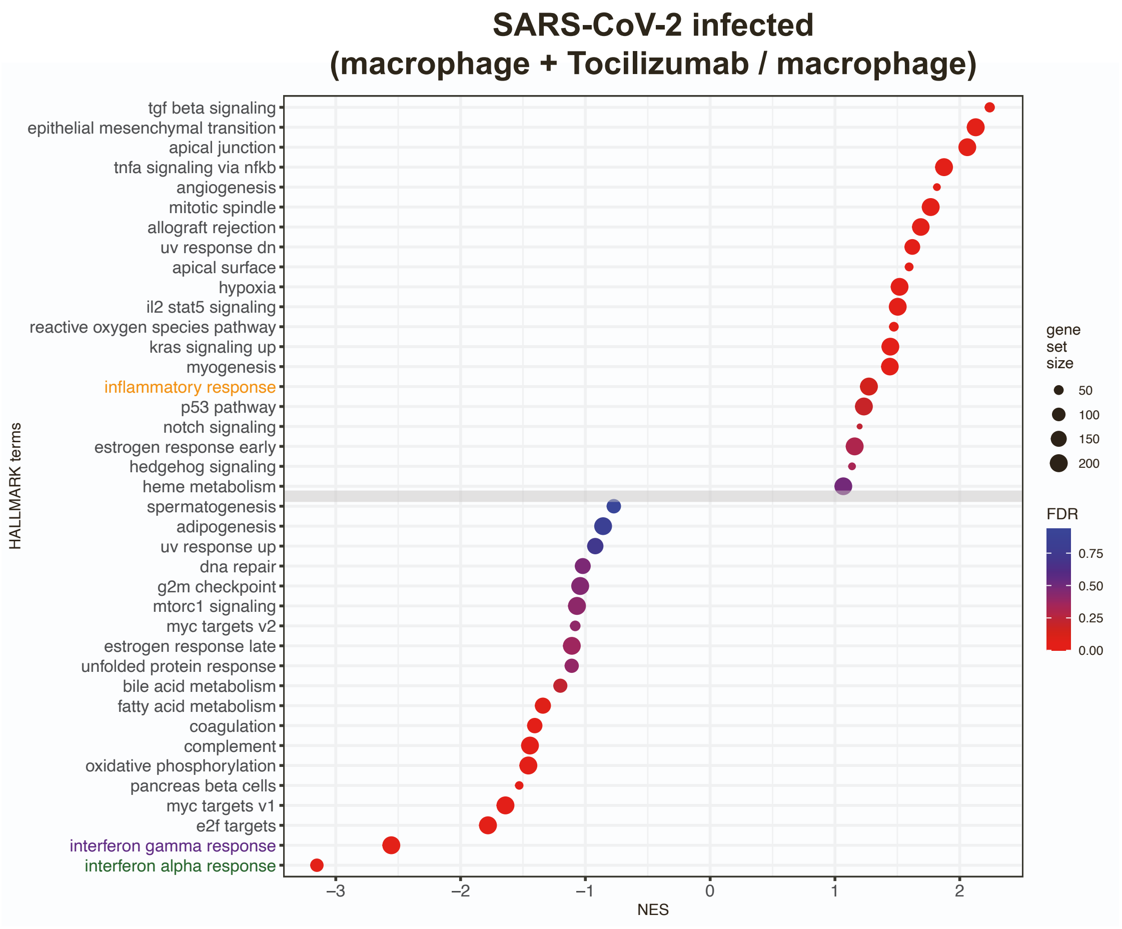
SARS-CoV-2 infected
(macrophage + Tocilizumab / macrophage)
HALLMARK terms
tgf beta signaling
epithelial mesenchymal transition
apical junction
tnfa signaling via nfkb
angiogenesis
mitotic spindle
allograft rejection
uv response dn
apical surface
hypoxia
il2 stat5 signaling
reactive oxygen species pathway
kras signaling up
myogenesis
inflammatory response
p53 pathway
notch signaling
estrogen response early
hedgehog signaling
heme metabolism
spermatogenesis
adipogenesis
uv response up
dna repair
g2m checkpoint
mtorc1 signaling
myc targets v2
estrogen response late
unfolded protein response
bile acid metabolism
fatty acid metabolism
coagulation
complement
oxidative phosphorylation
pancreas beta cells
myc targets v1
e2f targets
interferon gamma response
interferon alpha response
−3
−2
−1
0
1
2
NES
gene set size
50
100
150
200
FDR
0.75
0.50
0.25
0.00

(B)

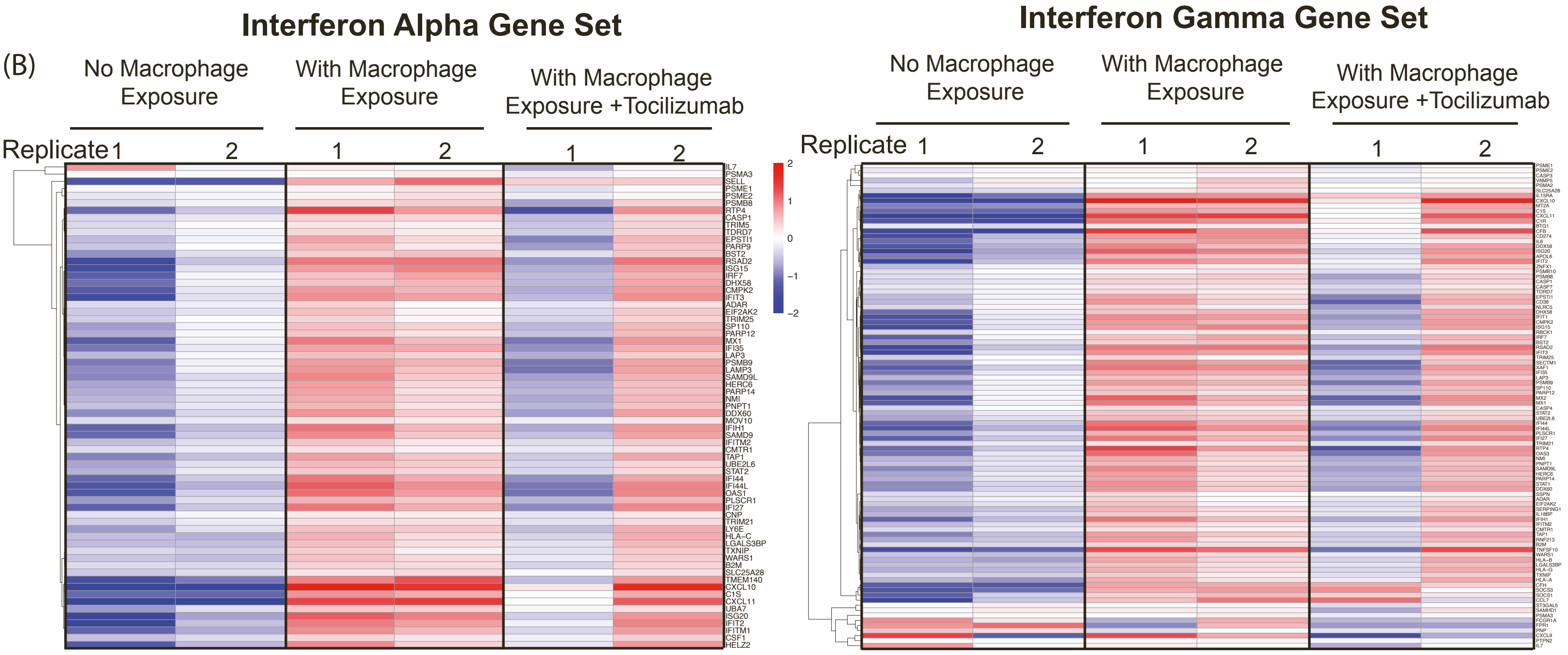
Interferon Alpha Gene Set
No Macrophage Exposure
With Macrophage Exposure
With Macrophage Exposure +Tocilizumab
Replicate 1
2
1
2
1
2
2
1
0
−1
−2
Interferon Gamma Gene Set
No Macrophage Exposure
With Macrophage Exposure
With Macrophage Exposure +Tocilizumab
Replicate 1
2
1
2
1
2

Figure S9

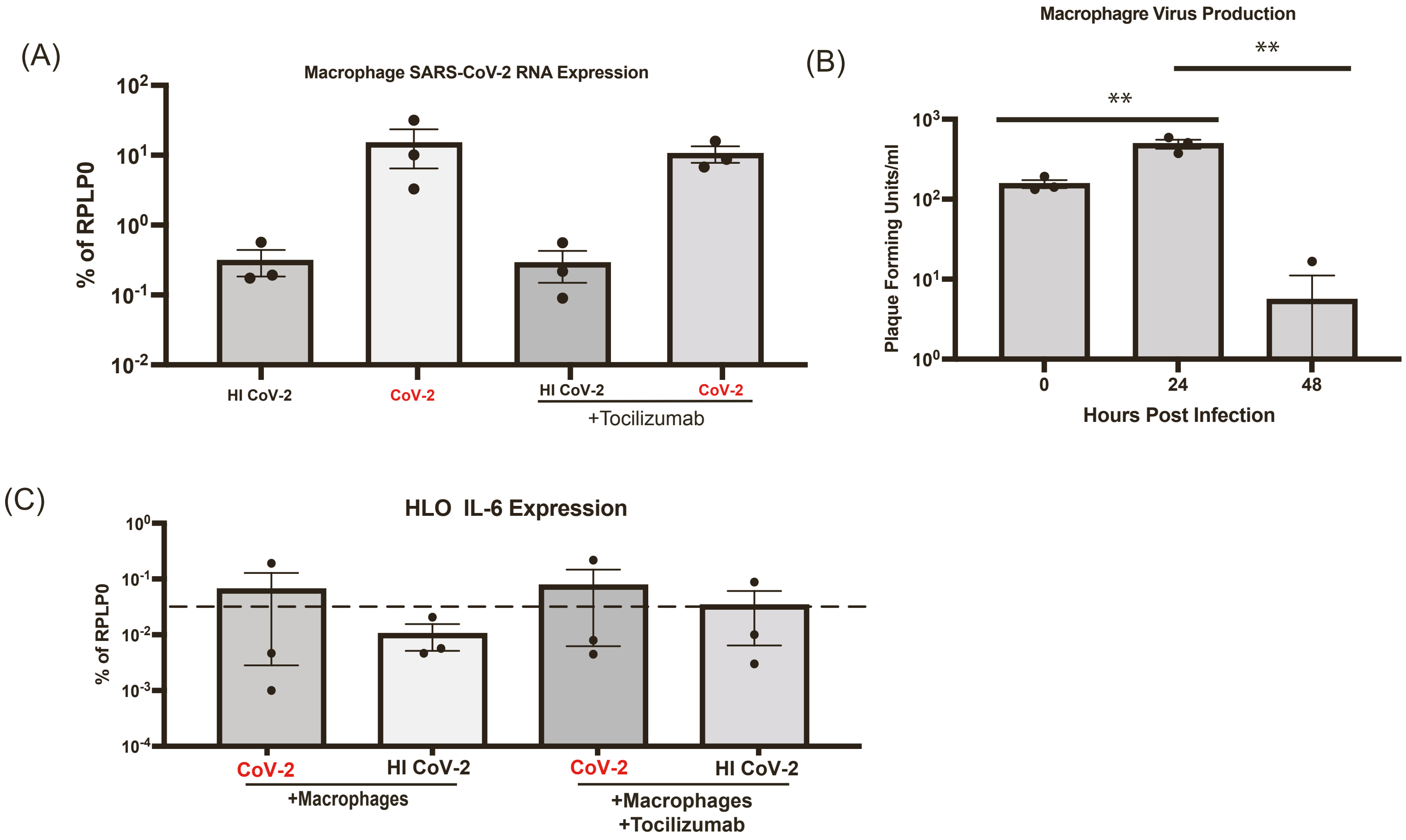

(A)
Macrophage SARS-CoV-2 RNA Expression
% of RPLP0
10^2
10^1
10^0
10^-1
10^-2
HI CoV-2
CoV-2
HI CoV-2
CoV-2
+Tocilizumab
(B)
Macrophagre Virus Production
**
**
Plaque Forming Units/ml
10^3
10^2
10^1
10^0
0
24
48
Hours Post Infection
(C)
HLO IL-6 Expression
% of RPLP0
10^0
10^-1
10^-2
10^-3
10^-4
CoV-2
HI CoV-2
+Macrophages
CoV-2
HI CoV-2
+Macrophages
+Tocilizumab

Table S1: Cell counts of populations identified based on reads mapped to the SARS-CoV-2 genome

Cell Line	Virus	Reads mapped to the SARS-CoV-2 genome		
		0-1	2-10	>10
H1	SARS-CoV-2	439	40	8
H1	HI SARS-CoV-2	533	7	2
1016	SARS-CoV-2	3387	511	56
1016	HI SARS-CoV-2	4329	3	0

Table S2: Cell counts for clusters identified in HLOs exposed to live SARS-CoV-2

Cluster	Cell Line	Positive for SARS-CoV-2 reads	Negative for SARS-CoV-2 reads
Hepatocyte-like cells 1	1016	209	2538
	H1	30	213
Hepatocyte-like cells 2	1016	198	583
	H1	12	188
Cholangiocyte-like cells	1016	160	1208
	H1	6	132

Table S3: Sequences for primers used in this study

Gene	Forward	Reverse
RPLP0	gcagcatctacaaccctgaag	gcagacagacactggcaaca
SARS-CoV-2	gcctcttctcgttcctcatcac	agcagcatcaccgccattg
Albumin	accccacacgcctttggcacaa	cacacccctggaataagccgagct
HNF4a	catggccaagattgacaacct	ttcccatatgttcctgcatcag
IL-6	actcacctcttcagaacgaattg	ccatctttggaaggttcaggttg
MCP-1	cagcagcaagtgtcccaaag	gagtgagtgttcaagtcttcgg

Figure S1: SARS-CoV-2 infectious virus production in HLO cells, Related to Figure 1.

(A)HLOs generated from H1 iPSCs were dissociated and cultured in 12-well plates. Cell were infected with SARS-CoV-2 at a multiplicity of infection of 1. After a 1-hour absorption inoculum was removed and fresh media added to HLO cells, and this was considered 0 hours post infection. Media was collected and replaced with fresh media at the indicated time post infection. **(B)** Representative confocal images of HLOs infected with SARS-CoV-2 and stained for dsRNA and KRT17; scale bars, 100 μm.

Figure S2: Expression pattern of liver specific genes in HLOs, Related to Figure 2

UMAP visualization of the integrated scRNA-seq data from HLOs infected with SARS-CoV-2 or treated with heat inactivated virus. Each panel shows the normalized expression from the RNA assay for the gene labeled above the panel. SERPINA1= serpin family A member 1, ALB=albumin, ASGR1= asialoglycoprotein receptor 1, CYP3A4= cytochrome P450 family 3 subfamily A member 4, TF=transferrin, TTR= transthyretin

Figure S3: Integration of hPSC HLO scRNA-seq data with single nuclei transcriptomic data from SARS-CoV-2 infected patients, Related to Figure 2

UMAP visualization of the hPSC derived HLO scRNA-sequencing samples integrated with the sn-RNA-seq liver autopsy samples (A), or only the Hepatocytes and Cholangiocytes from the liver autopsy samples(B). Panel (C) shows expression of BST2,

IFI35, IFI6, and ISG15 in UMAP visualization of HLO data integrated with patient hepatocytes and Cholangiocytes (B).

Figure S4: Transcriptomic Response of HLOs to SARS-CoV-2 Infection, Related to Figure 2

HLOs generated from 1016 iPSCs were infected with SARS-CoV-2 and sequenced with 10x single-cell sequencing. **(A)** Heatmap of significantly differentially expressed genes (Adj. p-value $\leq$ 0.05) in infected HLO cells compared to cells in HLOs exposed to heat-inactivated SARS-CoV-2. **(B)** Dot-plot showing expression of top differentially expressed genes in each major cluster in infected HLO cells versus cells in HLOs exposed to HI SARS-CoV-2. **(C)** Expression pattern of top differentially expressed genes in relation to the number of SARS-CoV-2 genomes detected in the cell. The selection of the genes shown is detailed in Figure 2.

Figure S5: Transcriptomic Response of bystander cells to SARS-CoV-2 infection, Related to Figure 2. HLOs generated from H1 ESCs (ESC #1) or 1016 iPSCs (iPSC #1) were infected with SARS-CoV-2 and sequenced with 10x single-cell sequencing. Heatmaps show significantly differentially expressed genes (Adj. p-value $\leq$ 0.05) in cells within HLOs exposed to live Sars-CoV-2 that had one or zero reads mapping to the SARS-CoV-2 genome compared to cells in HLOs exposed to HI SARS-CoV-2.

Figure S6: Exposure to inactive viral particles is sufficient for induction of inflammatory signaling, while viral replication prolongs and amplifies response, Related to Figure 2

HLOs generated from 1016 iPSCs were infected with either live SARS-CoV-2, heat-inactivated SARS-CoV-2, or conditioned media (mock infected). Total RNA was isolated at either 48 hours post infection or 120 hours post infection and sequenced. (A) Heatmaps showing expression of genes identified as differentially expressed during infection in single cell sequencing (Figure 2) (B) Heatmap of the top 60 significantly upregulated genes (ranked by the DESeq2 statistic) between HLOs exposed to live SARS-CoV-2 and mock infected HLOs. (C) Heatmap of the top 60 significantly upregulated genes (ranked by the DESeq2 statistic) between HLOs exposed to heat-inactivated SARS-CoV-2 and mock infected HLOs.

Figure S7: Macrophage exposure promotes an inflammatory state during SARS-CoV-2 infection of hPSC derived HLOs, Related to Figure 4

HLOs generated from 1016 iPSCs were infected with SARS-CoV-2. At 48 hours post infection HLOs were transferred to a transwell system with or without HMacs (Figure 4A). Total RNA was isolated from HLOs at 120 hours post infection and sequenced. Heatmaps show the expression of the core enrichment genes from the GSEA gene sets enriched in infected HLO cultured with HMacs versus infected HLOs not cultured with HMacs (Figure 4B).

Figure S8: Blocking IL-6 signaling dampens the inflammatory response generated by co-culture with HMacs, Related to Figure 4

Gene set enrichment analysis (GSEA) comparing infected HLOs cultured with HMacs and tocilizumab to infected HLOs cultured with HMacs and DMSO. The top upregulated and downregulated gene sets are shown.

Figure S9: hPSC derived macrophages do not support sustained infection by SARS-CoV-2, Related to Figure 4

(A) RT-qPCR of SARS-CoV-2 nucleocapsid (NC) expression in HMacs 96 hours after the initiation of co-culture. **(B)** HMacs were infected with SARS-CoV-2 at a multiplicity of infection of 1. After a 1-hour absorption inoculum was removed and fresh media added, this was considered 0 hours post infection. Media was collected and replaced with fresh media at the indicated time post infection and infectious virus quantitated by plaque assay. **(C**) RT-qPCR of HLO IL-6 expression 96 hours after the initiation of HMac co-culture. Dotted line represents average IL-6 expression in HLOs not exposed to macrophages or tocilizumab (Figure 3A) For (A) and (C) Values are expressed as mean ± s.e.m. For (B) Values are expressed as mean ± s.d. (**= $p < 0.01$).

Table S1: Cell counts of populations identified based on reads mapped to the SARS-CoV-2 genome, Related to Figure 2B

Table S2: Cell counts for clusters identified in HLOs exposed to live SARS-CoV-2, Related to Figure 2B and 2E

Table S3: Sequences for primers used in this study, Related to Figure 1, Figure 3, Figure 4, and Figure S9
