# Supplementary material for: SARS-CoV-2 infection of human pluripotent stem cell-derived liver organoids reveals potential mechanisms of liver pathology
Source: iScience. 2022 Sep 16;25(10):105146. doi: 10.1016/j.isci.2022.105146 (PMC9477603; doi:10.1016/j.isci.2022.105146)
Supplement: Table S1. Cell counts of populations identified based on reads mapped to the SARSCoV-2 genome, related to Figure 2B [file mmc2.docx]

**Table S1:** Cell counts of populations identified based on reads mapped to the SARS-CoV-2 genome

| **Cell Line** | **Virus** | **Reads mapped to the SARS-CoV-2 genome** | | |
| --- | --- | --- | --- | --- |
|  |  | **0-1** | **2-10** | **>10** |
| H1 | SARS-CoV-2 | 439 | 40 | 8 |
| H1 | HI SARS-CoV-2 | 533 | 7 | 2 |
| 1016 | SARS-CoV-2 | 3387 | 511 | 56 |
| 1016 | HI SARS-CoV-2 | 4329 | 3 | 0 |
