# Supplementary material for: SARS-CoV-2 infection of human pluripotent stem cell-derived liver organoids reveals potential mechanisms of liver pathology
Source: iScience. 2022 Sep 16;25(10):105146. doi: 10.1016/j.isci.2022.105146 (PMC9477603; doi:10.1016/j.isci.2022.105146)
Supplement: Table S2. Cell counts for clusters identified in HLOs exposed to live SARS-CoV-2, related to Figures 2B and 2E [file mmc3.docx]

| **Cluster** | **Cell Line** | **Positive for SARS-CoV-2 reads** | **Negative for SARS-CoV-2 reads** |
| --- | --- | --- | --- |
| **Hepatocyte-like cells 1** | 1016 | 209 | 2538 |
|  | H1 | 30 | 213 |
| **Hepatocyte-like cells 2** | 1016 | 198 | 583 |
|  | H1 | 12 | 188 |
| **Cholangiocyte-like cells** | 1016 | 160 | 1208 |
|  | H1 | 6 | 132 |

**Table S2:** Cell counts for clusters identified in HLOs exposed to live SARS-CoV-2
