# Supplementary material for: SARS-CoV-2 infection of human pluripotent stem cell-derived liver organoids reveals potential mechanisms of liver pathology
Source: iScience. 2022 Sep 16;25(10):105146. doi: 10.1016/j.isci.2022.105146 (PMC9477603; doi:10.1016/j.isci.2022.105146)
Supplement: Table S3. Sequences for primers used in this study, related to Figures 1, 3, 4, and S9 [file mmc4.docx]

| Gene | Forward | Reverse |
| --- | --- | --- |
| RPLP0 | gcagcatctacaaccctgaag | gcagacagacactggcaaca |
| SARS-CoV-2 | gcctcttctcgttcctcatcac | agcagcatcaccgccattg |
| Albumin | accccacacgcctttggcacaa | cacacccctggaataagccgagct |
| HNF4a | catggccaagattgacaacct | ttcccatatgttcctgcatcag |
| IL-6 | actcacctcttcagaacgaattg | ccatctttggaaggttcaggttg |
| MCP-1 | cagcagcaagtgtcccaaag | gagtgagtgttcaagtcttcgg |

**Table S3:** Sequences for primers used in this study
